# Metabolic Versatility and Bioactive Metabolite Potential of *Pseudotabrizicola phototrophica* sp. nov., an Aerobic Anoxygenic Phototroph from Intertidal Sediments

**DOI:** 10.3390/md24100364

**Published:** 2026-10-19

**Authors:** Li Hu, Meng-Qi Ye, Jin-Hao Xu, Yu-Qi Ye, Xin-Yun Tan, Zong-Jun Du

**Affiliations:** 1 Marine College, Shandong University, Weihai 264209, China; 202367000011@sdu.edu.cn (L.H.); 202100700282@mail.sdu.edu.cn (J.-H.X.); yeyuqi2023@163.com (Y.-Q.Y.); 202237703@mail.sdu.edu.cn (X.-Y.T.); duzongjun@sdu.edu.cn (Z.-J.D.); 2 State Key Laboratory of Microbial Technology, Qingdao 266237, China; 3 Weihai Research Institute of Industrial Technology, Shandong University, Weihai 264209, China

**Keywords:** *Pseudotabrizicola phototrophica*, marine intertidal sediment, aerobic anoxygenic phototrophic bacteria (AAPB), polyhydroxybutyrate (PHB), carotenoids, secondary metabolites, genome mining

## Abstract

Marine microorganisms are prolific sources of bioactive natural products, yet bacteria from intertidal sediments remain underexplored. Here, we report the isolation and genomic characterization of *Pseudotabrizicola phototrophica* sp. nov. (strain L79^T^) from intertidal sediments of Xiaoshi Island, Yellow Sea, Weihai, China. Genome mining using antiSMASH 8.0 revealed seven biosynthetic gene clusters (BGCs) with pharmaceutical relevance, including a complete carotenoid (spirilloxanthin) cluster (confirmed by absorption spectroscopy at 367, 482, and 775 nm); an NRPS cluster for putative pyreudione-like compounds; a PKS cluster for 6-methylsalicylic acid, a bioactive polyketide; a terpene-precursor cluster for sodorifen; a butyrolactone-type cluster; a partial ectoine-related gene cluster; and a RiPP-like cluster potentially encoding a bufferin-like metallophore. Experimentally validated metabolic traits include polyhydroxybutyrate (PHB) biosynthesis (confirmed by TEM), thiosulfate oxidation (SOX), and assimilatory nitrate reduction to ammonium (ANRA). The identification of these BGCs—particularly the NRPS and PKS clusters linked to antimicrobial compounds—provides a genomic roadmap for future natural product discovery from this underexplored marine bacterium.

## 1. Introduction

Aerobic anoxygenic phototrophic bacteria (AAPB) represent a unique physiological group in marine ecosystems, characterized by light energy utilization through bacteriochlorophyll (BChl)-based reaction centers without producing oxygen, while remaining obligately dependent on oxygen for growth [1]. This fascinating metabolic strategy, known as aerobic anoxygenic photosynthesis, provides them with a significant ecological advantage by supplementing their heterotrophic energy metabolism with light-derived energy [2]. First described in the 1970s, AAPB have since been recognized as ubiquitous and abundant components of marine ecosystems, potentially accounting for up to 11% of the total microbial community in some oceanic regions [3,4]. Their distribution patterns across diverse aquatic environments, from open oceans to coastal waters, reflect adaptations to varying light and nutrient conditions [5,6]. It is important to note that, unlike anaerobic phototrophs, AAPB are obligate aerobes; they require oxygen for growth, and their phototrophic apparatus functions as an auxiliary energy source to supplement heterotrophic metabolism, rather than replacing respiration.

Marine microorganisms have emerged as a prolific and promising source of structurally unique and biologically active natural products, offering immense potential for pharmaceutical, nutraceutical, and cosmeceutical applications [7,8]. The discovery of novel marine bacteria, particularly those from underexplored habitats such as intertidal sediments, is critical for expanding the chemical diversity of marine-derived drug leads [9]. In this context, genome mining has become an indispensable tool, enabling the prediction of biosynthetic gene clusters (BGCs) that encode for the production of valuable secondary metabolites before their experimental isolation [10]. Carotenoids from marine bacteria, for instance, have attracted considerable attention for their significant antioxidant activities and potential applications in dietary supplements, cosmetics, and pharmaceuticals [11]. Similarly, polyhydroxybutyrate (PHB) produced by marine bacteria has emerged as a promising biodegradable and biocompatible alternative to conventional petroleum-derived plastics, with applications in medical implants and drug delivery carriers [12]. Genome mining has become an indispensable tool in marine natural product discovery, enabling the prediction of BGCs prior to experimental isolation [7,13]. Recent studies have demonstrated that marine bacteria from underexplored habitats harbor a wealth of novel BGCs with potential pharmaceutical applications [14]. This approach is particularly valuable for prioritizing strains for chemical investigation, thereby accelerating the discovery of novel bioactive compounds.

Marine intertidal sediments represent particularly dynamic and challenging environments that serve as important interfaces between terrestrial and marine ecosystems [15]. Characterized by regular fluctuations in physical and chemical parameters including temperature, salinity, oxygen availability, and light exposure, these sediments host complex microbial communities that drive critical biogeochemical cycles [16,17]. The microbial inhabitants of intertidal zones have developed sophisticated adaptive strategies to cope with these variable conditions, making them valuable sources of novel metabolic capabilities and ecological adaptations [18,19]. Particularly, intertidal sediments have been shown to harbor diverse AAPB populations that may contribute significantly to both carbon and nutrient cycling in these ecosystems [20,21].

Microbial metabolism of sulfur and nitrogen plays a crucial role in modulating sediment redox conditions and nutrient retention. The thiosulfate oxidation (SOX) system enables bacteria to oxidize reduced sulfur compounds, which can mitigate sulfide inhibition of organic matter degradation in sediments [22,23]. Likewise, assimilatory nitrate reduction to ammonium (ANRA) allows microorganisms to directly incorporate nitrogen into biomass [24,25].

The classification and phylogenetic diversity of AAPB have evolved significantly with advances in molecular techniques. The genus *Pseudotabrizicola* was recently established through a comprehensive reclassification of members previously assigned to the genus *Tabrizicola*, based on polyphasic taxonomic analyses that revealed distinct phylogenetic and phenotypic characteristics warranting separation at the genus level [26]. This reclassification highlights the ongoing refinement of alphaproteobacterial taxonomy as genomic data becomes more accessible. Members of the *Pseudotabrizicola* genus have been isolated from diverse habitats including saline lakes, sediments, and algal cultures, with several species identified as AAPB [27,28,29]. The presence of photosynthetic genes and BChl *a* production in these species suggests that phototrophy represents a significant ecological strategy within this genus, possibly contributing to their success in nutrient-limited environments.

The discovery and characterization of novel AAPB species from understudied environments like intertidal sediments remains crucial for advancing our understanding of microbial diversity, ecological function, and evolutionary adaptations, as well as for expanding the repertoire of marine-derived bioactive compounds [14]. Despite increased research attention in recent years, the full phylogenetic diversity and metabolic potential of AAPB, particularly those inhabiting sedimentary environments, remains incompletely explored [30]. In this study, we isolated strain L79^T^ from intertidal sediments of Xiaoshi Island, Yellow Sea (Weihai, China). Through polyphasic taxonomic analysis, we confirmed it as a novel *Pseudotabrizicola* species. The objectives of this study are (1) to taxonomically characterize strain L79^T^ and establish it as a new species; (2) to describe its genomic and physiological features, with a focus on its phototrophic and metabolic capabilities; and (3) to perform genome mining for biosynthetic gene clusters (BGCs) with biotechnological potential; and (4) to discuss the potential implications of these features for marine natural product discovery and biogeochemical cycling in intertidal sediments, while acknowledging the limitations of a culture-based study.

## 2. Results and Discussion

### 2.1. Taxonomic Characterization of Strain L79^T^

The taxonomic characterization of strain L79^T^ is presented systematically, including growth and morphological characteristics, chemotaxonomic traits, phylogenetic analysis, and species delineation.

#### 2.1.1. Growth Condition

**Figure 1:**
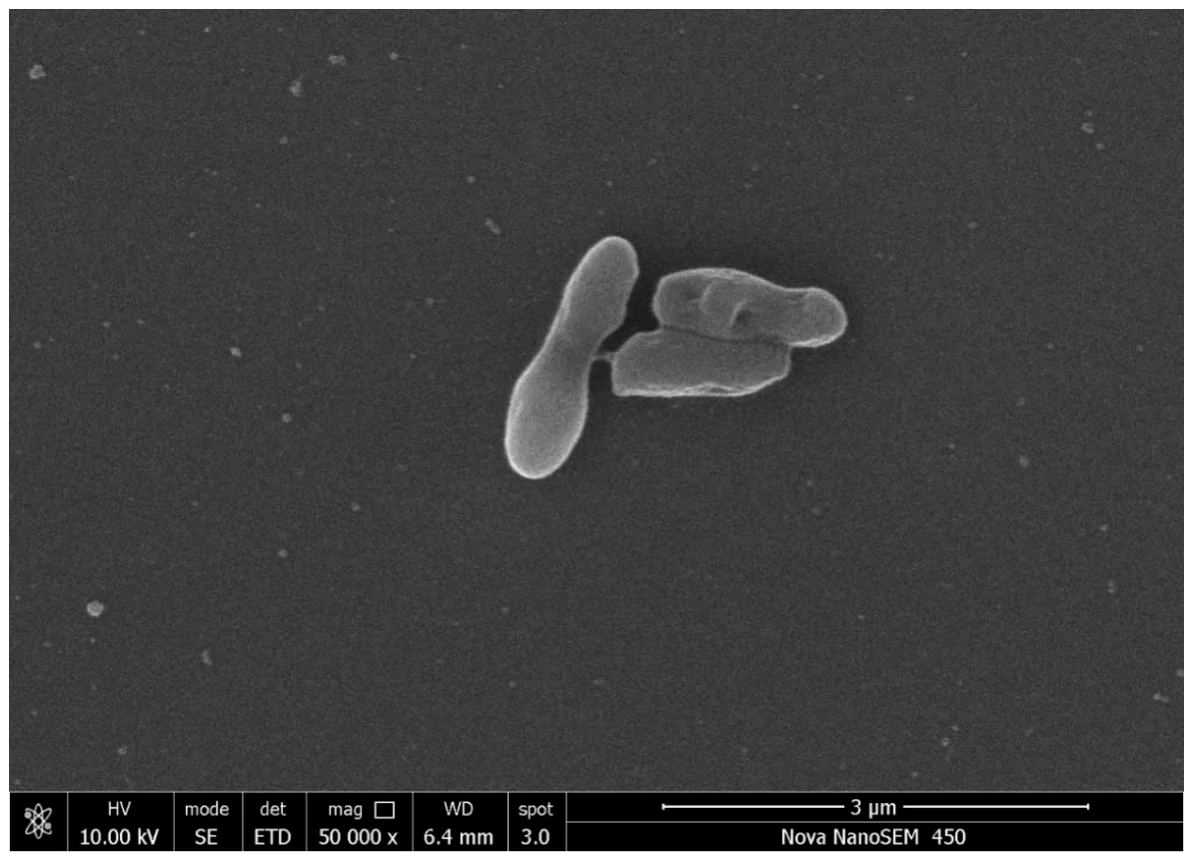
Scanning electron micrograph of strain L79^T^, showing its rod-shaped cellular morphology at 50,000× magnification. Colonies on Marine Agar 2216 are faint yellow, smooth, and circular (1.0–1.5 mm in diameter) after 3 days at 30 °C, and become opaque red after two weeks under light conditions).

Colonies of strain L79^T^ were faint yellow in color, smooth, and circular following 3 days of incubation at 30 °C on MA medium. Under the light condition, the colony color shifted to opaque red after two weeks. Cells of strain L79^T^ were Gram-stain negative and rod-shaped, exhibiting a width of 0.3–0.5 μm and a length of 0.8–1.5 μm (Figure 1). Growth occurred at 4–37 °C (optimum 25–30 °C), 0.5–4.0% NaCl (optimum 2.5% NaCl), and pH 6.5–9.0 (optimum pH7.0–7.5).

#### 2.1.2. Chemotaxonomic Traits

Chemotaxonomic analysis revealed that the respiratory quinone of strain L79^T^ was exclusively ubiquinone-10 (Q-10), which is consistent with members of the genus *Pseudotabrizicola*. Fatty acid profiling indicated that the dominant cellular fatty acids (>10%) were Summed Feature 8 (comprising C_18:1_ω7c and/or C_18:1_ω6c; 61.1%) and iso-C_18:0_ (13.8%). A full comparative analysis of fatty acid composition between strain L79^T^ and the reference strains *Pseudotabrizicola algicola* KCTC 72206^T^ and *Pseudotabrizicola sediminis* KCTC 72015^T^ is provided in Tables S1 and S2. The polar lipid profile consisted of phosphatidylglycerol (PG), phosphatidylethanolamine (PE), phosphatidylcholine (PC), and three unidentified lipids (L1–L3), as illustrated in Table S1. These chemotaxonomic traits are in accordance with those typical of the genus *Pseudotabrizicola*. Nevertheless, several distinct characteristics differentiated strain L79^T^ from related species, including differences in colony pigmentation (faint yellow vs. cream), optimal NaCl concentration (2.5% vs. 2.0% for *P. algicola* and 1.5% for *P. sediminis*), and the ability to reduce nitrate (positive vs. negative in *P. sediminis*), as summarized in Table S1, supporting its status as a novel species within the genus.

#### 2.1.3. Phylogenetic Analysis

**Figure 2:**
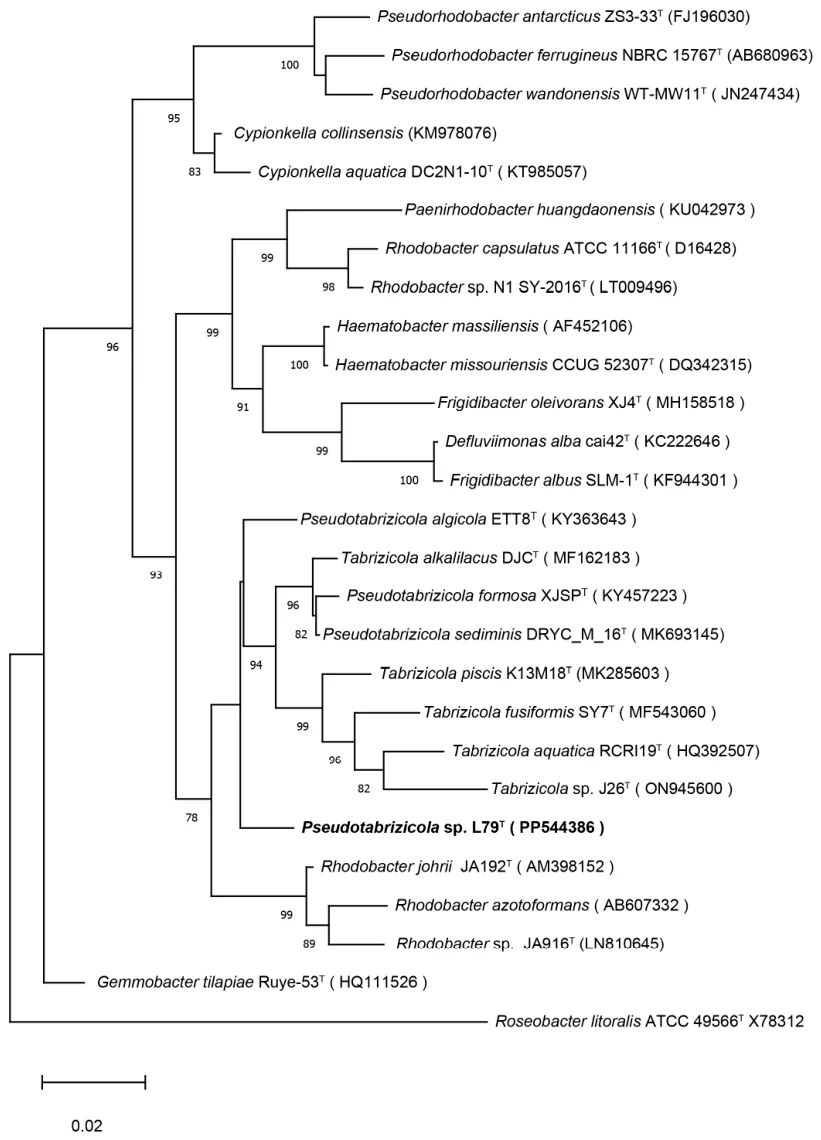
Maximum-likelihood phylogenetic tree based on 16S rRNA gene sequences, showing the phylogenetic position of strain L79^T^ among closely related species. The tree was constructed using sequences obtained from the EzBioCloud database. Bootstrap values (≥70%) are shown at branch nodes. Strain L79^T^ forms a distinct clade within the genus *Pseudotabrizicola* and exhibits the highest sequence similarity (98.0%) with *Pseudotabrizicola algicola* KCTC 72206^T^. The scale bar represents 0.02 substitutions per nucleotide position.

The near-complete 16S rRNA gene sequence of strain L79^T^ (1435 bp; GenBank accession number: PP544386) was obtained, and its draft genome sequence was extracted (JAZHDA010000000). The sequence of *P. algicola* KCTC 72206^T^ was found to be the most similar to that of strain L79^T^, with a 98.0% similarity. The 16S rRNA gene sequences of closely allied species were amassed from the EzBioCloud and employed to assemble the phylogenetic tree using the maximum-likelihood (ML) method. The tree included all four validly described species of the genus *Pseudotabrizicola* currently listed in LPSN (https://lpsn.dsmz.de/genus/pseudotabrizicola) accessed on 14 October 2026 (*Pseudotabrizicola algicola*, *Pseudotabrizicola alkalilacus*, *Pseudotabrizicola sediminis*, and *Pseudotabrizicola formosa*), ensuring comprehensive coverage of the genus. In accordance with the phylogenetic trees constructed from 16S rRNA gene sequences (Figure 2 and Figure S1), strain L79^T^ was closely related to *P. algicola*, and was in a separate clade within the genus *Pseudotabrizicola*. Furthermore, the protein phylogenetic tree, showing the evolutionary relationships of strain L79^T^ and some related type strains, indicated the strain was affiliated with the genus *Pseudotabrizicola* (Figure S2), which was consistent with the result of 16S rRNA gene phylogenetic analysis.

To validate the relationship between strain L79^T^ and related taxa, whole genome sequences of all available type strains of species belonging to *Pseudotabrizicola* were downloaded from NCBI (https://www.ncbi.nlm.nih.gov/) accessed on 17 October 2026. Subsequently, ANI and dDDH values with L79^T^ were calculated (Table S3). The ANI values between strain L79^T^ and its closest relatives (*P. algicola* KCTC 72206^T^, *P. sediminis* KCTC 72015^T^, *P. alkalilacus* KCTC 62173^T^, and *P. formosa* KCTC 52636^T^) range from 76.5% to 77.1%, all well below the 95–96% threshold for species delineation. Similarly, the dDDH values range from 22.5% to 23.1%, far below the 70% cutoff. Although the 16S rRNA gene sequence similarity with *P. algicola* is 98.0%, this value exceeds the commonly used 97% cutoff but cannot be used alone for species demarcation; the ANI and dDDH values, which are well below the thresholds, provide the decisive evidence for classifying L79^T^ as a novel species. Collectively, these genomic and phylogenetic data, together with the phenotypic differences summarized in Table S1, unequivocally support the classification of strain L79^T^ as a novel species within the genus *Pseudotabrizicola*.

### 2.2. Substrate Utilization Profile of Strain L79^T^

Strain L79^T^ failed to exhibit either autotrophic or heterotrophic growth under anaerobic conditions. The inability to grow autotrophically or anaerobically underscores an obligate dependence on aerobic respiration coupled with photoheterotrophy. This metabolic profile is consistent with a lifestyle adapted to the oxic–anoxic interfaces of sediments, where light, organic carbon, and oxygen availability fluctuate.

Strain L79^T^ showed positive reactions towards a diverse suite of carbon substrates including β-methyl-D-glucose, N-acetyl-D-glucosamine, D-fucose, D-mannitol, D-fructose-6-phosphate, methyl pyruvate, α-hydroxybutyric acid, and 2-nitrophenyl β-D-galactopyranoside, while showing a negative reaction towards sorbitol (Table S2). This specific substrate utilization profile is consistent with related *Pseudotabrizicola* species [27,29,31]. The negative response to sorbitol further refines this profile.

The differential distribution of genes involved in osmoregulation (e.g., ectoine-related genes), light harvesting (carotenoid biosynthesis genes), and carbon utilization (e.g., specific sugar transporters) among *Pseudotabrizicola* species likely underpins their adaptation to distinct niches, such as saline lakes (*P. alkalilacus*), algal cultures (*P. algicola*), and intertidal sediments (strain L79^T^, *P. phototrophica*). At the genus level, the core genome provides metabolic flexibility (e.g., phototrophy), while the species-specific accessory genome may confer specialization to particular carbon substrates.

### 2.3. Genomic Analysis

#### 2.3.1. Genomic Features and Genus-Level Architecture

The draft genome of strain L79^T^ is 3,950,661 bp in size, assembled into 58 contigs with an N50 of 363,760 bp. Genome completeness was estimated at 98.7% with contamination of 0.4% as assessed by CheckM. The genome of strain L79^T^ comprises 3880 genes, including 3804 protein-coding genes and 54 RNA genes—specifically, 3 rRNA genes, 48 tRNA genes, and 3 non-coding RNAs. The genomic DNA G + C content was determined to be 62.3 mol% (Table S3). Consistent with other members of the genus *Pseudotabrizicola*, strain L79^T^ contains three rRNA operons. The draft genome of strain L79^T^ contains three rRNA genes. However, because the genome is assembled into 58 contigs, the exact number of ribosomal operons cannot be determined with certainty. The presence of three rRNA genes is consistent with other members of the genus *Pseudotabrizicola*, but the possibility of additional rRNA operons in unassembled regions cannot be excluded. It has been established that the number of rRNA gene copies in bacterial genomes correlates with ecological strategy and growth rate: bacteria adapted to nutrient-rich environments that support rapid growth typically possess higher rRNA gene copy numbers (averaging approximately 5.5 copies), whereas slow-growing organisms from oligotrophic or stressful environments often have fewer copies (averaging around 1.4 copies) [32]. The presence of three rRNA genes in strain L79^T^ suggests an intermediate metabolic strategy [32], though the incomplete genome assembly precludes a definitive conclusion regarding rRNA operon copy number and its requirement for prolonged incubation under optimal conditions (e.g., specific temperature and extended culture duration) to develop its characteristic red pigmentation. While rRNA operon copy number has been widely used as an indicator of life history traits such as resource responsiveness and growth rate [33,34], recent studies caution that its application as a sole indicator of overall ecological strategy may be overly simplistic [35]. The intermediate copy number observed in L79^T^ is therefore consistent with an adaptation to the fluctuating nutrient conditions of its intertidal sediment habitat, balancing rapid resource utilization with survival efficiency in a variable environment. This genomic trait may reflect an adaptation to the fluctuating nutrient conditions of its intertidal sediment habitat.

The genomic G + C content of strain L79^T^ is 62.3 mol%, which falls within the range observed for other *Pseudotabrizicola* species (62.9–64.4 mol%). Its intermediate rRNA operon copy number (three) is consistent with a moderately slow growth strategy.

**Figure 3:**
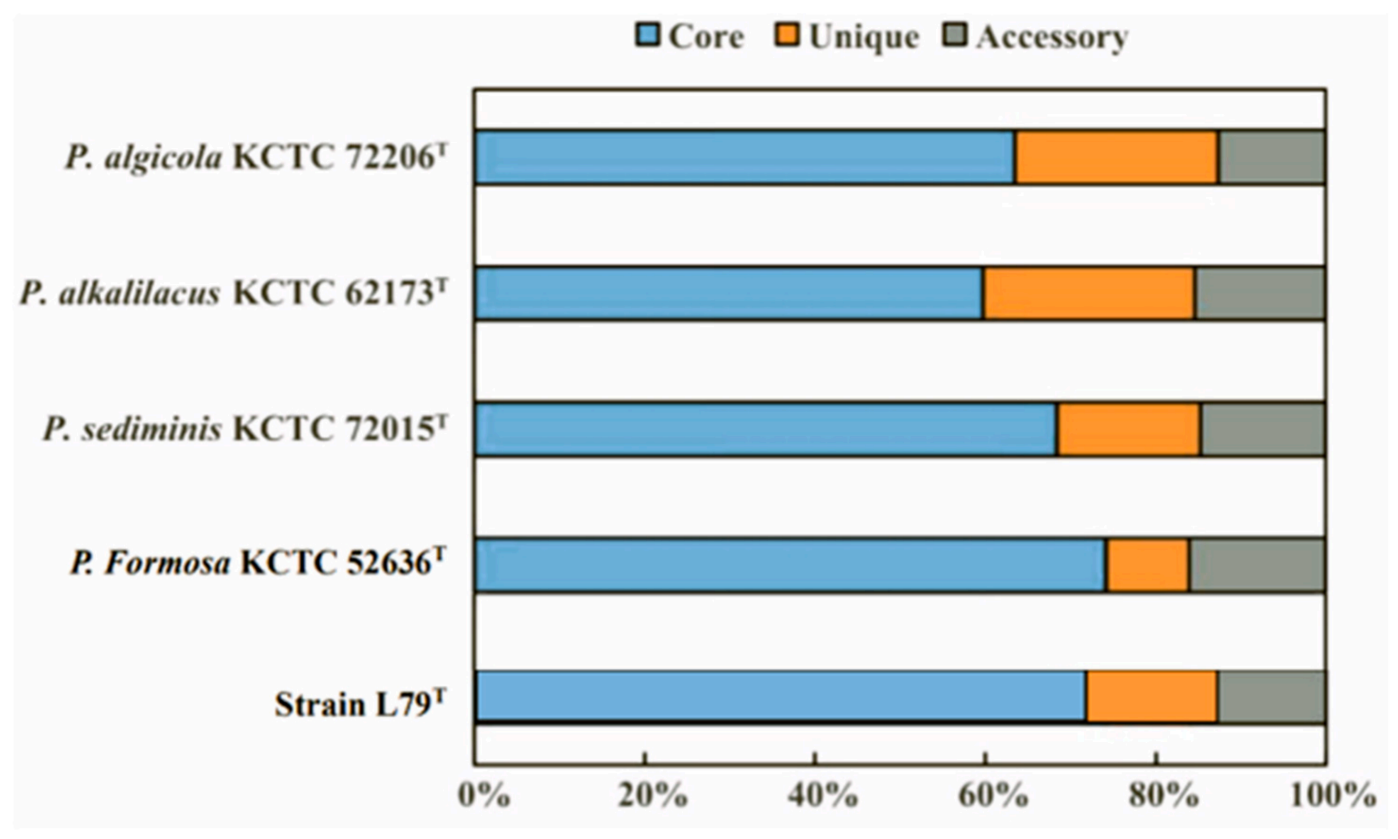
Comparative genomic analysis of five *Pseudotabrizicola* species, including strain L79^T^. The bar plot depicts the distribution of gene categories in each genome, highlighting a conserved core genome of 2570 genes (59.8–74.2% of individual genomes) essential for central metabolic and information processing functions.

Comparative pan-genome analysis of five *Pseudotabrizicola* species (including strain L79^T^, *P. formosa* KCTC 52636^T^, *P. sediminis* KCTC 72015^T^, *P. alkalilacus* KCTC 62173^T^, and *P. algicola* KCTC 72206^T^) revealed a core genome of 2570 genes, accounting for 59.8–74.2% of each individual genome (Figure 3). The core genome is enriched in housekeeping functions (amino acid, energy, and nucleotide metabolism; translation, replication, and repair). Notably, the photosynthetic reaction center genes (*pufL*, *pufM*) and bacteriochlorophyll biosynthesis genes are part of the core genome, indicating that anoxygenic photosynthesis is a conserved trait of the genus. These genes are responsible for light harvesting and energy conversion and are distinct from the carotenoid biosynthesis genes, which are located in a separate BGC (Cluster 1) and are discussed in Section 2.3.2.

The accessory genome (genes present in 2–4 strains) is enriched in carbohydrate metabolism, cofactor/vitamin biosynthesis, and xenobiotic degradation, contributing to metabolic plasticity. Unique genes (present in only one strain) are associated with drug resistance, lipid metabolism, membrane transport, and signal transduction, and may underpin species-specific adaptations. This genomic architecture—a stable core combined with flexible accessory genes—likely facilitates niche differentiation among *Pseudotabrizicola* species. Specific metabolic pathways (e.g., PHB synthesis, thiosulfate oxidation, nitrate assimilation) are discussed in detail in the following section (Section 2.3.2).

A detailed comparison of key metabolic gene distribution among the three *Pseudotabrizicola* genomes for which complete data are available (L79^T^, *P. algicola*, and *P. sediminis*) is provided in Table 1. This analysis reveals that genes for anoxygenic photosynthesis (*pufL*, *pufM*, *bch* cluster), PHB synthesis (*phbB*, *phaC*, *phaE*, *phaZ*, *phaR*), and assimilatory nitrate reduction (*nas*, *nir*) are present in all three strains. In contrast, the sox cluster and assimilatory sulfate reduction genes (*cys*) show differential distribution, being present only in L79^T^ and one of the two reference strains.

#### 2.3.2. Core Metabolic Pathways

**Figure 4:**
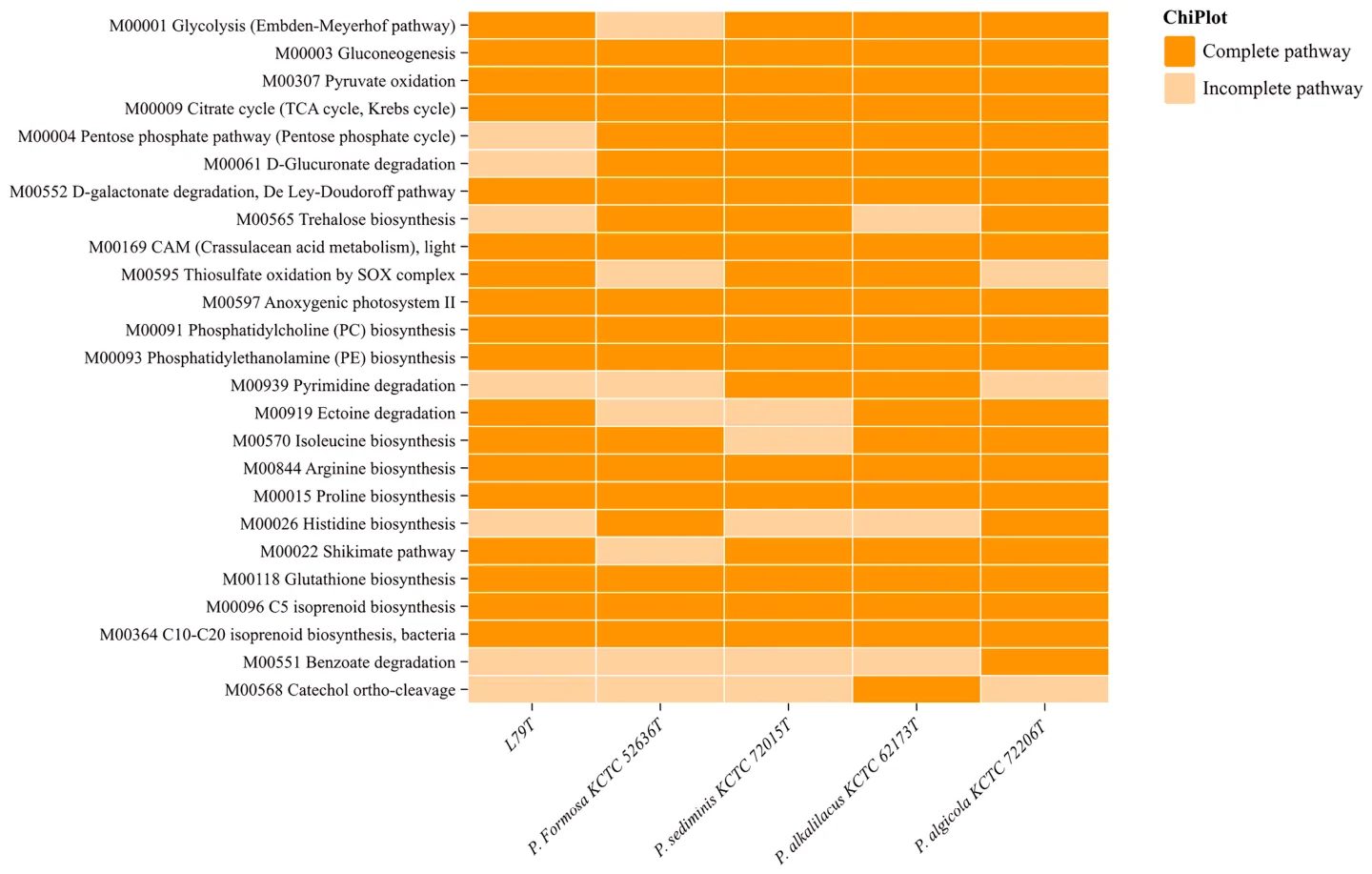
Genomic annotation reveals a suite of complete metabolic pathways in strain L79^T^ involved in carbon metabolism, sulfur cycling, and nitrogen assimilation.

Genomic annotation of strain L79^T^ (BlastKOALA and KEGG analysis) revealed a suite of complete metabolic pathways (Figure 4) involved in carbon metabolism, sulfur cycling, and nitrogen assimilation.

#### Carbon Metabolism: Phototrophy and Polyhydroxybutyrate (PHB) Storage

Central carbohydrate metabolic pathways, including glycolysis (M00001, M00002) and gluconeogenesis (M00003), were complete, indicating metabolic versatility in energy acquisition and carbon utilization. This metabolic plasticity is characteristic of many aerobic anoxygenic phototrophs, enabling them to adapt to fluctuating nutrient conditions in their native environments [36,37]. Notably, consistent with the genus’s core genomic trait, L79^T^ possessed a complete anoxygenic photosystem II (M00597), encoding the PufL and PufM subunits of the photosynthetic reaction center [38]. The conservation of this system across the genus underscores the ecological importance of phototrophy as an adaptive strategy. The presence of these genes supports the classification of *Pseudotabrizicola* as a genus of aerobic anoxygenic phototrophic bacteria and suggests that light-enhanced energy acquisition provides a selective advantage in their respective niches [5,37].

Transmission electron microscopy (TEM) was performed to examine the intracellular membrane organization of strain L79^T^. The images revealed some vesicular and lamellar membrane structures, but well-defined photosynthetic membranes (e.g., thylakoid stacks) were not resolved due to insufficient image quality. Therefore, these images are not shown. The presence of a complete set of photosynthetic genes (*pufL*, *pufM*) (Table 1) and the absorption spectrum of bacteriochlorophyll *a* provide functional evidence for phototrophy, regardless of the ultrastructural details.

A key species-specific carbon trait was the complete polyhydroxyalkanoate (PHA, primarily PHB) metabolic pathway. Genomic annotation revealed a substantial genetic repertoire for PHA metabolism in strain L79^T^ (Table S4). The genome contains *phbB*, encoding acetoacetyl-CoA reductase, which catalyzes the reduction in acetoacetyl-CoA to (R)-3-hydroxybutyryl-CoA. Additionally, both *phaC* and *phaE* genes were identified, encoding the two subunits of the poly[(R)-3-hydroxyalkanoate] polymerase complex that catalyzes the final polymerization step in PHB biosynthesis [39]. The genome also contains *phaZ*, encoding poly(3-hydroxybutyrate) depolymerase, completing the genetic framework for both PHB synthesis and mobilization. This genetic capacity for PHB synthesis and mobilization allows cells to store excess carbon during periods of abundance and mobilize it during scarcity [40]. The genome also encodes phaR, a polyhydroxyalkanoate synthesis repressor protein, which plays a role in regulating PHB metabolism by repressing PHB synthase expression under non-permissive conditions and binding to accumulated PHB granules to derepress synthesis during storage conditions [41]. The presence of phaR indicates a regulatory mechanism for PHB metabolism in strain L79^T^. Comparative analysis shows that the PHB synthesis genes (*phbB*, *phaC*, *phaE*) and the depolymerase gene (*phaZ*) are present in all three *Pseudotabrizicola* genomes (Table 1), indicating that PHB accumulation and mobilization are genus-wide traits. Notably, the regulatory gene *phaR* is also present in all three strains, suggesting that the regulatory mechanism of PHB metabolism is conserved within the genus. The identification of a complete and regulated PHB biosynthetic pathway in strain L79^T^ has significant biotechnological implications. PHB is a biodegradable and biocompatible thermoplastic polyester that has attracted considerable interest for biomedical applications, including surgical sutures, wound dressings, and drug delivery systems [42]. Marine bacteria have emerged as promising sources for PHB production, with several studies reporting PHB accumulation in marine *Vibrio*, *Streptomyces*, and *Erythrobacter* species [43,44,45,46]. The coexistence of PHB synthesis with phototrophic energy capture may reduce the metabolic burden of PHB accumulation, potentially enhancing production yields compared to heterotrophic PHB producers, making this strain a promising candidate for further optimization studies.

**Figure 5:**
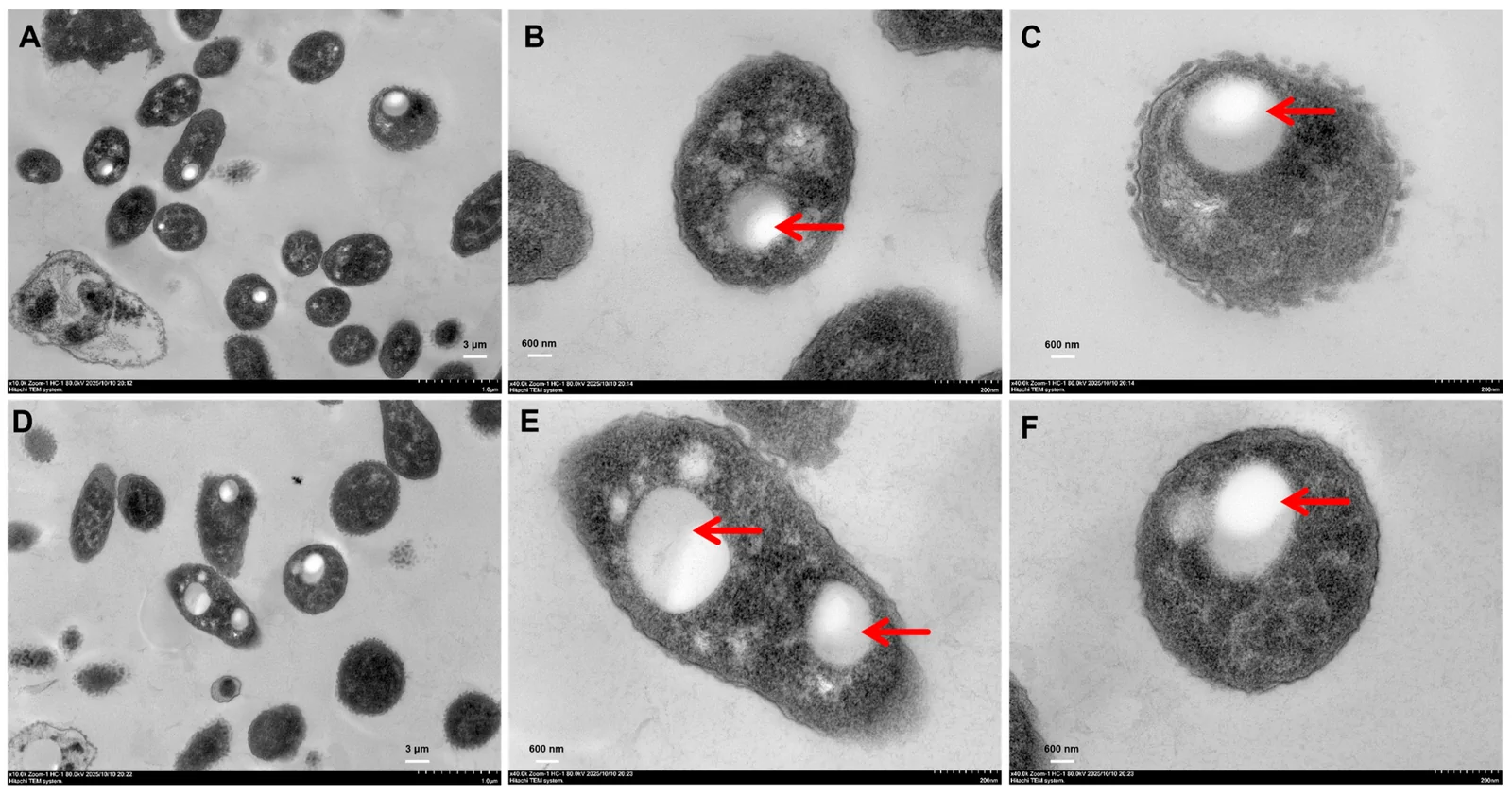
Transmission electron micrographs of ultrathin sections of strain L79^T^. (**A**,**D**) General views of cells with a 3 μm scale bar. (**B**,**C**) Higher-magnification views of individual cells from panel (**A**) with a 600 nm scale bar. (**E**,**F**) Higher-magnification views of individual cells from panel (**D**) with a 600 nm scale bar. Electron-transparent granules (indicated by arrows) are consistent with PHB inclusion bodies. Electron-dense regions represent the cytoplasm. No well-defined thylakoid stacks were observed.

The metabolic context for PHB precursor supply appears well-established through complementary pathways. The complete glycolysis (M00001) and tricarboxylic acid cycle (M00009) pathways provide acetyl-CoA precursor, while fatty acid biosynthesis pathways (M00082) further demonstrate robust acetyl-CoA metabolism in this strain. Supporting this genomic evidence, transmission electron microscopy of ultrathin sections revealed numerous electron-transparent granular structures within the cytoplasm of strain L79^T^ (Figure 5). These structures exhibited morphological characteristics consistent with PHB inclusion bodies commonly observed in diverse bacterial species [47]. However, quantitative analysis of PHB content and polymer-specific characterization are required in future studies to confirm the extent of PHB accumulation.

Furthermore, the strain harbors intact isoprenoid biosynthesis pathways (M00096, M00364), which are critical for producing carotenoids and quinones—compounds integral to photoprotection and electron transport, respectively [48,49]. These pathways, conserved across *Pseudotabrizicola* species, are involved in light energy capture and mitigating photo-oxidative damage. Additionally, strain L79^T^ contains complete pathways for the synthesis of phosphatidylcholine (M00091) and phosphatidylethanolamine (M00093), essential phospholipid components of bacterial membranes that contribute to membrane stability under environmental stress [50].

#### Photoprotective Pigmentation and Biosynthetic Gene Clusters

The genomic foundation for the phototrophic lifestyle of strain L79^T^ was established by the identification of key metabolic gene clusters. It should be noted that the chlorophyll biosynthesis and light-harvesting complex (LHC) genes are functionally associated with light harvesting and are distinct from the carotenoid biosynthesis genes discussed below, which are responsible for photoprotection and accessory light harvesting. antiSMASH 8.0 analysis revealed predicted secondary metabolite biosynthetic gene clusters (BGCs) as summarized in Table 2. This repertoire encompasses diverse compound classes with documented or predicted pharmaceutical and biotechnological relevance, including a terpene cluster for carotenoid biosynthesis, an NRPS cluster for putative pyreudione-like compounds, a PKS cluster for putative 6-methylsalicylic acid analogs, a terpene-precursor cluster for sodorifen, a butyrolactone-type cluster, a partial ectoine-related gene cluster, and a RiPP-like cluster potentially encoding a bufferin-like metallophore. The similarity scores reported by antiSMASH (28–82%) indicate varying degrees of homology to known BGCs. High similarity (e.g., 82% for Cluster 1) suggests that the encoded pathway is likely to produce compounds closely related to the known cluster, whereas low similarity (e.g., 28% for Cluster 7) indicates that the encoded products may be structurally novel and difficult to predict based on homology alone. Therefore, all predicted compounds should be regarded as putative and require experimental validation.

**Figure 6:**
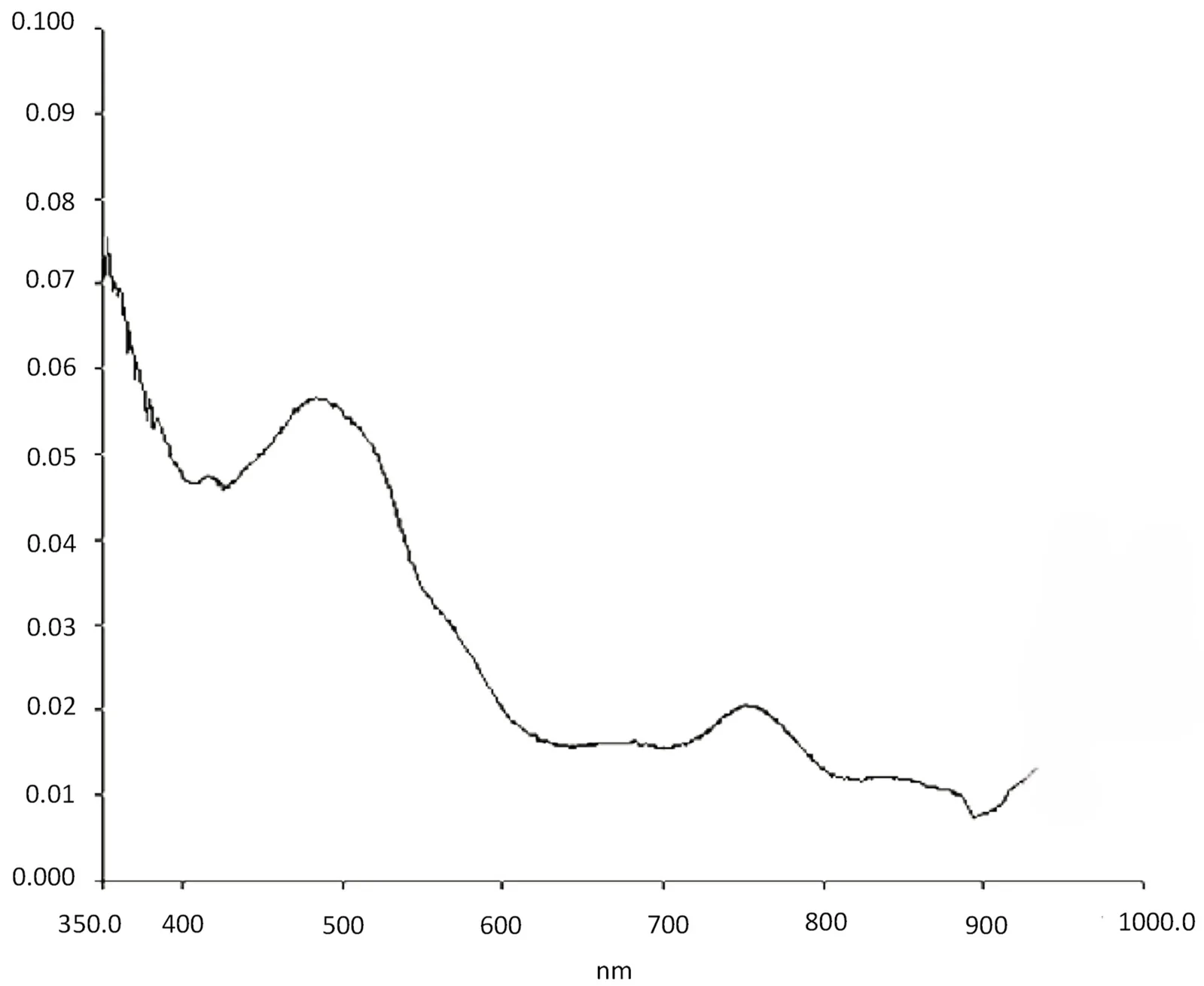
Absorption spectrum of an acetone/methanol extract from strain L79^T^ shows characteristic peaks at 367, 482, and 775 nm, confirming the presence of bacteriochlorophyll *a* and carotenoids of the spirilloxanthin series. This profile is consistent with the photosynthetic apparatus of aerobic anoxygenic phototrophic bacteria.

Terpene cluster (Cluster 1): Carotenoid biosynthesis. The terpene cluster in strain L79^T^ exhibits 82% similarity to a known carotenoid biosynthesis gene cluster and is predicted to encode a complete set of genes for the spirilloxanthin biosynthesis pathway, including *crtE*, *crtB*, *crtI*, *crtC*, *crtD*, and *crtF* (Table 3). This gene composition is characteristic of aerobic anoxygenic phototrophic bacteria that produce spirilloxanthin-series carotenoids [51], and is consistent with the observed absorption spectrum (Figure 6). Carotenoids from marine bacteria have been widely recognized for their significant antioxidant activities, with potential applications in the nutraceutical, cosmetic, and pharmaceutical industries [10,52]. The confirmed production of carotenoids by strain L79^T^, supported by both genomic and spectroscopic evidence, positions this strain as a promising source for carotenoid-based bioactive compounds.

NRPS cluster (Cluster 2): Pyreudione biosynthesis. The NRPS cluster in strain L79^T^ shows 74% similarity to the pyreudione biosynthesis gene cluster. Pyreudiones are a family of bacterial alkaloids produced by a minimalistic nonribosomal peptide synthetase, and have demonstrated antibacterial activity against *Mycobacterium* species, including *M. aurum* and *M. vaccae* [53]. The biosynthetic pathway involves a single-module NRPS that incorporates an unusual β-keto acid starter unit, making it an attractive target for combinatorial biosynthesis and structural diversification. The presence of this cluster in strain L79^T^ suggests the genetic capacity to produce putative pyreudione-like derivatives.

PKS cluster (Cluster 3): 6-Methylsalicylic acid biosynthesis. The PKS cluster in strain L79^T^ shows 60% similarity to a 6-methylsalicylic acid (6-MSA) biosynthesis gene cluster. 6-MSA is a bioactive polyketide with documented antimicrobial and antifungal activities [54]. It also serves as a key intermediate in the biosynthesis of antifungal agents and anticancer compounds, including the promising drug candidate 6-MSA derivatives. The presence of this cluster indicates the genomic basis for producing putative 6-MSA analogs with potential therapeutic applications.

Terpene-precursor cluster (Cluster 4): Sodorifen biosynthesis. The terpene-precursor cluster in strain L79^T^ shows 49% similarity to a sodorifen biosynthesis gene cluster. Sodorifen is a non-canonical C16 terpenoid with a unique bicyclooctene framework, produced by *Serratia plymuthica* and *Pseudomonas* species [55,56]. While its biological activity remains underexplored, sodorifen and its derivatives have attracted attention for their volatile-mediated ecological functions and potential applications as bioactive fumigants or plant growth promoters. The presence of this cluster in strain L79^T^ implies the potential for biosynthesis of putative terpenoid derivatives with potential agrochemical or pharmaceutical applications.

Butyrolactone cluster (Cluster 5): Putative butyrolactone-type signaling molecule. The butyrolactone cluster in strain L79^T^ shows 46% similarity to a known butyrolactone BGC in the MIBiG database. However, the most similar known cluster in the MIBiG database is associated with (2R,3S,4S)-5-fluoro-2,3,4-trihydroxypentanoic acid in *Streptomyces* sp. MA37, which is structurally distinct from acyl-homoserine lactones [57]. Therefore, the product of Cluster 5 cannot be confidently assigned, and this cluster may encode a butyrolactone-type signaling molecule rather than specifically an acyl-homoserine lactone. Acyl-homoserine lactones (AHLs) are quorum-sensing signal molecules that regulate virulence factor production, biofilm formation, and secondary metabolite biosynthesis in Gram-negative bacteria [58]. While AHLs themselves are not therapeutic agents, their biosynthetic enzymes and regulatory circuits are attractive targets for anti-virulence drugs and quorum-sensing inhibitors. Experimental characterization is required to determine the actual product of Cluster 5.

Ectoine cluster (Cluster 6): Partial ectoine-related gene cluster and ectoine utilization potential. The ectoine cluster in strain L79^T^ shows 43% similarity to a known ectoine biosynthesis gene cluster. This cluster contains the ectoine synthase gene (*ectC*), which catalyzes the final cyclization step in ectoine biosynthesis. However, a complete ectoine biosynthetic pathway typically requires additional genes, including *ectA* (diaminobutyrate acetyltransferase) and *ectB* (diaminobutyrate aminotransferase) [59,60]. A search of the L79^T^ genome for these additional genes did not identify any homologs of *ectA* or *ectB*. Therefore, the presence of only *ectC* suggests that strain L79^T^ may not possess a complete ectoine biosynthetic pathway, and the potential for ectoine production remains uncertain.

Interestingly, the genome of strain L79^T^ contains several genes associated with ectoine utilization, including homologs of ectoine hydrolase (*eutABC*/*doeAB*) and ectoine transporters (*EhuABCD*). The ectoine hydrolase gene is the first enzyme required for ectoine catabolism, hydrolyzing ectoine to *N*-α-acetyl-L-2,4-diaminobutyrate [61]. Ectoine transporters, such as the TRAP-type TeaABC system identified in *Halomonas elongata*, mediate the uptake of exogenous ectoine and hydroxyectoine with high affinity [62]. This suggests that strain L79^T^ may be able to take up exogenous ectoine and utilize it as a compatible solute or carbon/nitrogen source, rather than synthesizing it de novo. In the intertidal sediment environment, ectoine released by other microorganisms could serve as a readily available osmoprotectant, and the ability to scavenge exogenous ectoine may provide a competitive advantage under fluctuating salinity and osmotic stress. However, the functional role of these ectoine utilization genes remains to be experimentally validated. Further studies are required to determine whether strain L79^T^ can indeed transport and catabolize ectoine, and to what extent this contributes to its osmotic stress tolerance.

RiPP-like cluster (Cluster 7): Putative bufferin-like metallophore. The RiPP-like cluster in strain L79^T^ shows 28% similarity to a known RiPP BGC in the MIBiG database. The presence of Pfam domains DUF2282 (precursor peptide) and DUF692 (multinuclear non-heme iron-dependent oxidase, MNIO), suggests that this cluster may encode a member of the bufferin family of ribosomal peptide metallophores. Bufferins are a recently described and widespread family of RiPP natural products involved in bacterial adaptation to metal stress, particularly copper homeostasis [63]. They contain rare 5-thiooxazole modifications installed by MNIO enzymes and function by complexing excess metal ions to enhance bacterial growth under metal stress conditions. The biosynthetic machinery of bufferins is distinct from that of lanthipeptides, and the low similarity (28%) suggests that this cluster may encode a novel bufferin-like metallophore. This annotation highlights the potential of strain L79^T^ for producing novel RiPP-based metal-chelating compounds, which are of increasing interest for biotechnological and pharmaceutical applications. Experimental characterization is required to determine the actual product of Cluster 7.

Summary and future directions. The identification of seven BGCs in strain L79^T^—particularly the NRPS, PKS, and RiPP-like clusters—provides a genomic roadmap for future natural product discovery. These clusters represent a largely untapped reservoir of secondary metabolites with potential antibacterial, antifungal, antioxidant, and cytoprotective activities. Future studies should prioritize: (1) metabolomic profiling of strain L79^T^ under different cultivation conditions to identify expressed metabolites; (2) heterologous expression of selected BGCs in suitable host systems to enable compound isolation and structure elucidation; and (3) bioactivity-guided fractionation to evaluate the pharmacological potential of the produced metabolites. Such efforts will not only validate the genomic predictions but also accelerate the discovery of novel marine-derived therapeutics. The BGCs identified in this study are predicted based on bioinformatic analysis; their actual expression and the production of bioactive compounds have not been experimentally confirmed. Therefore, the pharmaceutical potential of strain L79^T^ remains hypothetical and requires future chemical and pharmacological validation.

The functional expression of this genomic potential was confirmed through phenotypic analysis. Spectral characterization of acetone/methanol extracts showed an absorption profile with maxima at 367 nm, 482 nm, and 775 nm (Figure 6), providing direct evidence for the synthesis of bacteriochlorophyll *a* (BChl *a*) and carotenoids [1]. The specific spectral signatures, including a blue-shifted BChl *a* peak and a carotenoid pattern indicative of the spirilloxanthin series, align with typical features of aerobic anoxygenic phototrophic bacteria [4,64,65]. This correlation between genomic prediction and phenotypic validation confirms the existence of a functional photosynthetic apparatus in strain L79^T^.

Carotenoids, encoded by the identified cluster, serve the dual role of light-harvesting and protecting the photosynthetic reaction centers from photo-oxidative damage [66]. The observed high carotenoid-to-BChl ratio may be an adaptation to the fluctuating light regime of the intertidal zone, providing enhanced protection during high-light exposure while maintaining harvesting efficiency during low-light periods [67,68]. This inducible photoprotection is a known physiological mechanism for maintaining metabolic continuity under light stress [69,70].

Beyond their role in the producing organism, spirilloxanthin-series carotenoids are of interest to humans due to their potent antioxidant activities, including singlet oxygen quenching and free radical scavenging, which may contribute to reducing oxidative stress and preventing related diseases. They are therefore promising candidates for applications in nutraceuticals, cosmetics, and pharmaceuticals.

Genomic annotation revealed that strain L79^T^ possesses a complete thiosulfate oxidation pathway (SOX system; M00595) and a complete assimilatory sulfate reduction pathway (M00176), but lacks the dissimilatory sulfate reduction pathway. This genetic repertoire suggests a metabolic strategy focused on thiosulfate oxidation and sulfur assimilation rather than anaerobic respiration [22]. To experimentally validate the SOX system functionality, we conducted assays monitoring sulfate production from thiosulfate. After incubation, significant barium sulfate precipitation was observed in bacterial suspensions treated with barium chloride, while no precipitation occurred with ferric chloride treatment (Table 4). These results confirm thiosulfate oxidation to sulfate, supporting the genomic prediction that strain L79^T^ utilizes the complete SOX system for sulfur oxidation.

The presence of a complete SOX system enables thiosulfate oxidation, which can influence sediment redox conditions. By oxidizing reduced sulfur compounds, strain L79^T^ may mitigate sulfide inhibition of carbon degradation processes [22,23]. Additionally, the presence of a complete assimilatory sulfate reduction pathway was confirmed through experiments showing decreased sulfate concentrations over extended incubation periods, as indicated by reduced barium sulfate precipitation after 10 days compared to day 5 (Table 4). This demonstrates the strain’s capacity for sulfate incorporation into organic sulfur compounds, essential for biosynthesis of methionine, cysteine, and other sulfur-containing cellular components [71]. The coexistence of complete SOX and assimilatory sulfate reduction pathways in strain L79^T^ represents a sulfur metabolic network that allows the bacterium to oxidize reduced sulfur compounds while simultaneously incorporating sulfate into biomass. Such capabilities may be advantageous in intertidal sediments where sulfur compounds show spatial and temporal heterogeneity due to fluctuating redox conditions [72,73]. The distribution of sulfur metabolism genes varies among species: the sox cluster is present in L79^T^ and *P. sediminis* but absent in *P. algicola*, while the assimilatory sulfate reduction genes (*cys*) are present only in L79^T^ among the three strains examined (Table 1). This differential distribution suggests that sulfur metabolic strategies may vary within the genus.

Genomic analysis revealed a complete SOX system, suggesting the genetic potential for thiosulfate oxidation. Experimental validation confirmed the strain’s ability to produce sulfate from thiosulfate (see Section 4.5.1 for method). However, the strain’s tolerance to sulfide and its capacity to oxidize sulfide directly were not tested in this study and remain to be investigated. This metabolic configuration may allow the bacterium to oxidize reduced sulfur compounds under the tested conditions, but direct evidence for its ecological relevance in natural environments is lacking. The absence of dissimilatory sulfate reduction genes further refines our understanding of the ecological niche occupied by strain L79^T^. Unlike sulfate-reducing bacteria that contribute to anaerobic sulfide production [74], strain L79^T^ appears specialized for oxidative sulfur transformations, potentially functioning in concert with sulfate-reducing partners in syntrophic relationships within sediment microbial communities [75].

#### Assimilatory Nitrate Reduction to Ammonium (ANRA)

**Figure 7:**
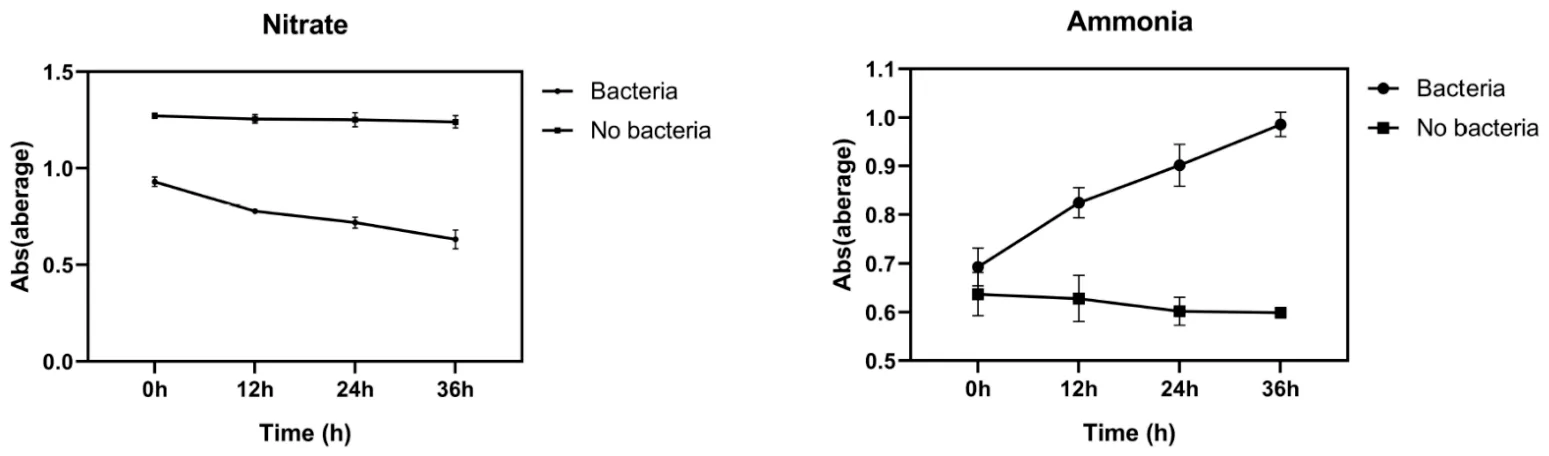
Time-course profiles of nitrate consumption and ammonium accumulation during growth of strain L79^T^, confirming its capability for assimilatory nitrate reduction to ammonium.

Assimilatory nitrate reduction to ammonium (ANRA) is a common metabolic pathway in many bacteria. Its presence in strain L79^T^ is noted here as part of the comprehensive metabolic profile. Genomic analysis revealed a complete ANRA pathway (M00531), including assimilatory nitrate reductase (Nas) and nitrite reductase (Nir) [24]. Experimental validation confirmed that after 48 h of incubation, nitrate concentration decreased while microbial biomass nitrogen increased (Figure 7), demonstrating the capability of strain L79^T^ to perform ANRA. The *nas* and *nir* genes are present in all three *Pseudotabrizicola* genomes examined (Table 1), indicating that ANRA is a conserved metabolic trait within the genus. Therefore, the ability to assimilate nitrate is not a unique feature of L79^T^ but rather a shared characteristic of *Pseudotabrizicola* species. This pathway allows the direct incorporation of nitrogen into cellular biomass, supporting growth and biosynthesis.

Although nitrate concentrations in the isolation site were not measured, this nitrogen retention strategy may be beneficial in nitrogen-limited environments. However, direct evidence for its ecological advantage is lacking and requires future studies.

The metabolic properties of strain L79^T^ appear well-suited to the fluctuating conditions of intertidal sediments. Aerobic anoxygenic phototrophy allows it to supplement heterotrophic energy metabolism with light-derived energy, providing a competitive advantage when organic carbon is limiting. The ability to synthesize PHB enables carbon storage during periods of excess and mobilization during scarcity. The complete SOX system and ANRA pathway may help mitigate sulfide toxicity and retain nitrogen, respectively, under the dynamic redox conditions characteristic of intertidal sediments. These traits may have been shaped by the selective pressures of the marine environment, favoring versatile energy acquisition and stress tolerance.

### 2.4. Global Distribution of the Genus Pseudotabrizicola

**Figure 8:**
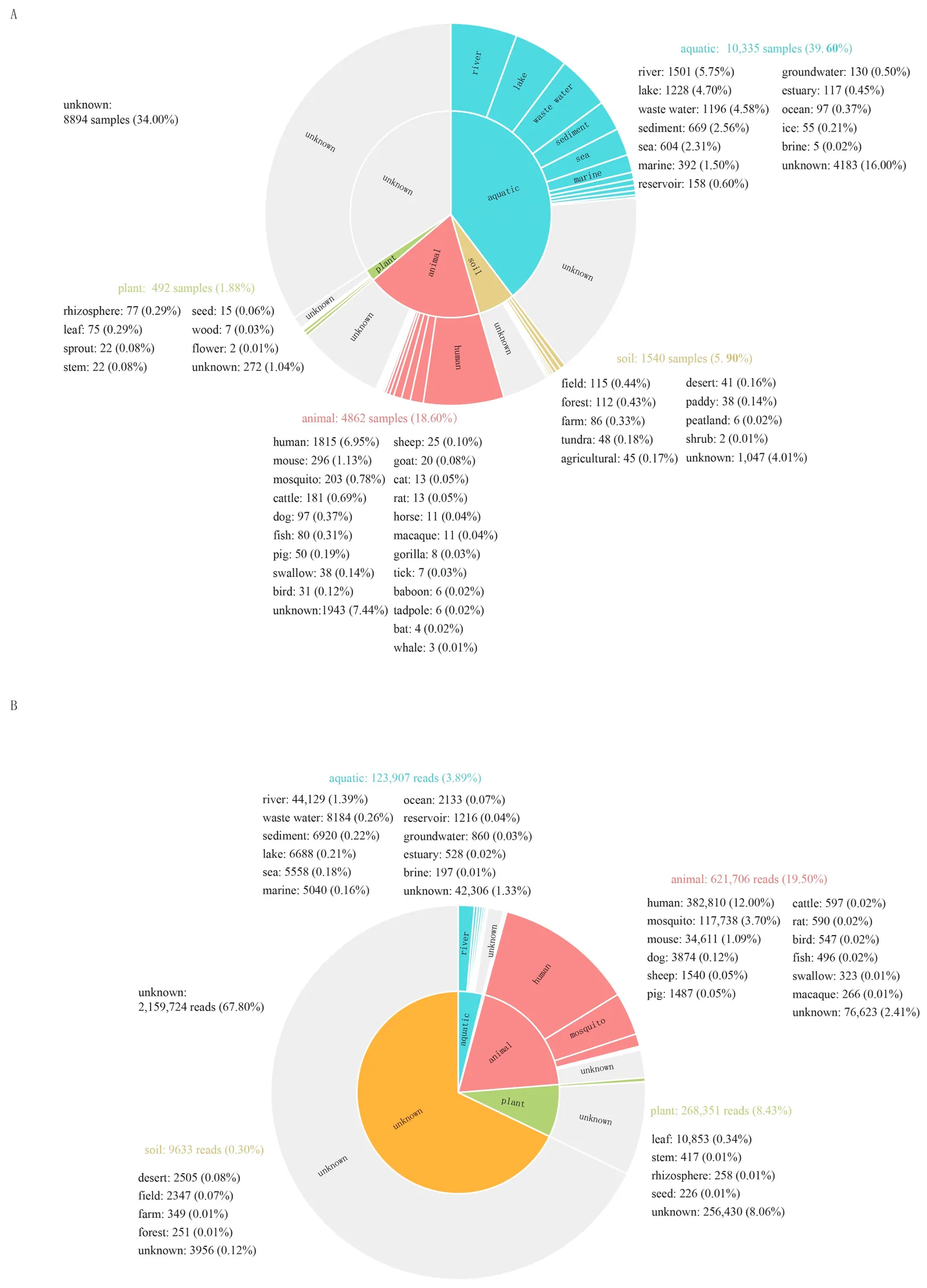
Global biogeographic distribution of the genus *Pseudotabrizicola* analyzed using the MAP database (v3). (**A**) Sample counts and (**B**) sequencing read proportions across major habitat types demonstrate its widespread occurrence in aquatic, animal-associated, soil, and plant-related environments.

Comprehensive analysis of the global distribution of the genus *Pseudotabrizicola* was conducted using the MAP database and pipeline (MAPv3) (https://microbeatlas.org/) accessed on 17 October 2026. The representative sequence was identified in 26,123 samples from 4822 projects, with a total of 3,183,321 sequencing reads mapped to this OTU, demonstrating its widespread occurrence across diverse ecosystems. Biogeographic distribution analysis revealed that *Pseudotabrizicola* members inhabit various environments, including aquatic, animal-associated, soil, and plant-related habitats (Figure 8). Sample prevalence data showed the highest occurrence in aquatic environments (10,335 samples, 39.60%), particularly in river (1501 samples, 5.75%), lake (1228 samples, 4.70%), wastewater (1196 samples, 4.58%), and sediment habitats (669 samples, 2.56%). This distribution pattern aligns with the isolation of strain L79^T^ from intertidal sediments and reflects adaptations to both freshwater and saline ecosystems [15,19].

However, quantitative analysis of sequencing reads revealed a contrasting pattern. The highest read abundance was observed in animal-associated environments (621,706 reads, 19.50%), with particularly strong signals in the human microbiome (382,810 reads, 12.00%) and mosquito guts (117,738 reads, 3.70%). The disproportionately high read abundance assigned to human-associated samples likely reflects well-documented biases in public metagenomic databases, where human microbiome studies are vastly over-represented, and reference database contamination can lead to misclassification of human reads [76,77]. To mitigate such biases in this analysis, we applied a minimum read abundance threshold (>100 reads per OTU) and relied on taxonomy assignments with high confidence scores as implemented in the MAP pipeline. The confirmed presence in marine sediments (6920 reads, 0.22% of total reads), albeit low, is more likely to reflect its genuine ecological niche.

The aquatic environments, which contained the most samples, accounted for a comparatively lower proportion of total reads (123,907 reads, 3.89%). Within these aquatic systems, the highest read abundances were detected in rivers (44,129 reads, 1.39%) and wastewater (8184 reads, 0.22%). Reads from marine sediments (6920 reads, 0.22%)—the isolation source of strain L79^T^—were quantifiable, confirming the presence of the genus in this habitat [18].

A high read abundance was detected in plant-related environments (268,351 reads, 8.43%), predominantly from unspecified plant niches. This, coupled with the substantial representation in aquatic systems, aligns with the genus’s genomic potential for phototrophy and utilization of plant-derived organic matter [18,27]. In contrast, soil environments (9633 reads, 0.30%) presented the lowest read abundance among the major biomes.

The differential distribution patterns between sample occurrence and read abundance highlight the complex ecology of *Pseudotabrizicola*. While widely distributed across environments (high sample prevalence), its population density (read abundance) varies significantly, suggesting niche-specific adaptations [78,79]. This pattern is consistent with the concept of microbial “core” and “flexible” genomic features, where conserved phototrophic capabilities provide a foundation, while accessory genes may enable niche specialization [80].

## 3. Implications for Marine Natural Product Discovery

The application of genome mining approaches has revolutionized the discovery of marine natural products by enabling the prediction of biosynthetic gene clusters and their associated compound classes prior to experimental isolation [13]. The identification of seven BGCs in strain L79^T^, including a complete carotenoid cluster and multiple polyketide and other specialized metabolite clusters, demonstrates the utility of this approach for assessing the biosynthetic potential of newly isolated marine bacteria.

Among these, the NRPS cluster and the PKS cluster are of particular interest for drug discovery, as the predicted compound classes have documented antimicrobial activities [53,54]. A partial ectoine-related gene cluster was also identified, though its role remains uncertain and requires further investigation. Marine microorganisms have been recognized as prolific sources of structurally diverse and pharmacologically active natural products, and the discovery of novel species from underexplored habitats such as intertidal sediments is critical for expanding the chemical diversity of marine-derived drug leads. The BGCs identified in strain L79^T^ warrant further investigation through metabolomics, heterologous expression, or bioactivity-guided fractionation to elucidate the structures and biological activities of the corresponding metabolites. This study thus provides a foundation for future natural product discovery efforts targeting this underexplored marine bacterium.

Limitations of genome mining. It is important to acknowledge that genome mining predicts BGCs based on sequence homology and does not guarantee that these clusters are expressed under laboratory conditions. Many BGCs are silent or poorly expressed, and the compounds they encode may not be produced in detectable amounts. Furthermore, the similarity scores reported by antiSMASH (28–82%) indicate varying degrees of homology to known clusters; low similarity (e.g., 28% for the RiPP-like cluster) suggests that the encoded products may be novel and difficult to predict. Therefore, the BGCs identified in this study should be regarded as a genomic roadmap for future chemical investigation rather than a definitive inventory of produced metabolites. Experimental validation through metabolomics, heterologous expression, and bioactivity-guided fractionation is essential to confirm the predicted compounds.

Strain L79^T^ can be distinguished from its closest relatives, *P. algicola* and *P. sediminis*, by several phenotypic and genomic features. Phenotypically, it differs in colony pigmentation (faint yellow vs. cream), optimal NaCl concentration (2.5% vs. 2.0% and 1.5%), and nitrate reduction ability (positive vs. negative in *P. sediminis*). Genomically, L79^T^ possesses a unique combination of BGCs (NRPS, PKS, and RiPP-like clusters) and a complete SOX system, which are absent or differentially distributed in the reference strains. These differences support its classification as a novel species and highlight its distinct metabolic potential.

## 4. Materials and Methods

### 4.1. Sample Collection, Bacterial Isolation, and Cultivation

Xiaoshi Island, Yellow Sea, situated in Weihai, China, has been officially recognized as a marine ecological protection zone, highlighting its importance as a significant continuous ecosystem in the region [15]. Sediment samples were collected in May 2021 at a depth of 5–20 cm (122°1′ E, 37°31′ N). For bacterial isolation, sediment samples were homogenized with sterile seawater (was prepared by autoclaving natural seawater at 121 °C for 20 min) and serially diluted to 10^−5^, Aliquots (100 µL) of each dilution were spread onto Marine Agar 2216 (MA; Qingdao Hope Bio-Technology Co., Ltd., Qingdao, China) and plates were incubated at 33 °C for 2 weeks, A faint yellow colony (strain L79^T^) was obtained through purification (Purification was performed by repeatedly streaking a single colony onto fresh MA plates three times) and stored at −80 °C in 15% (*v*/*v*) glycerol supplemented with 1% (*w*/*v*) sterile artificial seawater [17]. Reference strains *Pseudotabrizicola algicola* KCTC 72206^T^ and *Pseudotabrizicola sediminis* KCTC 72015^T^ were cultured under the same conditions for comparative analysis.

### 4.2. The 16S rRNA Gene and Genome Sequencing and Phylogenetic Analysis

The PCR amplification of strain L79^T^’s 16S rRNA gene utilized universal primers 27F and 1492R [81]. The sequence was cloned into the pMD18-T vector (Takara Bio Inc., Kusatsu, Shiga, Japan) for complete 16S rRNA gene sequencing. Genomic DNA was extracted with a DNA kit (Takara) and sequenced on the Illumina NovaSeq 6000 platform at Beijing Novogene Bioinformatics Technology Co., Ltd, Beijing, China. Raw reads were quality-filtered using fastp (version 0.20.0) and assembled using SOAPdenovo v2.04. Genome completeness and contamination were assessed with CheckM (version 1.1.3); genomes with >95% completeness and <5% contamination were selected for phylogenomic analysis.

For the 16S rRNA-based phylogenetic tree, 27 closely related sequences were retrieved from EzBioCloud. Multiple sequence alignment was performed using MAFFT v7.475. The best-fit substitution model (GTR + CAT) was selected using ModelFinder as implemented in IQ-TREE v1.6.12, with 1000 ultrafast bootstrap replicates. A second tree was constructed using FastTree v2.1.10 with the K3Pu + F + I + G4 model for comparison. Trees were visualized using iTOL.

For the phylogenomic tree, GTDB-Tk v2.1.1 was used to extract a concatenated alignment of 120 single-copy marker proteins (bacterial set from GTDB release R207). The maximum-likelihood tree was reconstructed using IQ-TREE v1.6.12 with the YEAST + F + I + G4 model, and 1000 bootstrap replicates. *Roseobacter litoralis* ATCC 49566^T^ was used as an outgroup.

### 4.3. Genomic DNA Sequencing, Assembly, Annotation, and Genome Comparison

The 16S rRNA gene sequences were compared in the EzBioCloud and NCBI databases to confirm the taxonomic status. ANI [82] and dDDH [83] values between strain L79^T^ and others were calculated based on genome sequences. Genome annotation was performed using the KEGG database to study metabolic pathways [84]. The antiSMASH database predicted secondary metabolites, while the CARD database analyzed antibiotic resistance [85,86]. Metabolic pathways and environmental adaptation mechanisms were studied based on KEGG annotations. Photosynthetic genes *pufML* were identified in the genome using specific primers and SnapGene v. 7.2 [87].

Comparative genomic analysis of five *Pseudotabrizicola* strains (including L79^T^, *P. formosa* KCTC 52636^T^, *P. sediminis* KCTC 72015^T^, *P. alkalilacus* KCTC 62173^T^, and *P. algicola* KCTC 72206^T^) was performed using BPGA v1.3 with default parameters [88]. Core genes were defined as those present in 100% of the strains, accessory genes as those present in 2–4 strains, and unique genes as those found in only one strain.

### 4.4. Growth Conditions and Biochemical Characterization

Strain L79^T^ was characterized phenotypically, biochemically, and chemotaxonomically after cultivation on MA at 37 °C for 3 days. Experiments were conducted in triplicate unless stated otherwise. Cell characteristics were observed using light microscopy (Nikon E600) and scanning electron microscopy (FEI NovaNanoSEM450, FEI Company, Hillsboro, OR, USA). Gram-staining was performed following Gabani’s method [89]. Gliding motility was assessed on 0.3% agar MA plates based on previous protocols [90]. The growth range and optimal temperature were determined by observing colonies on modified MA at temperatures from to 40 °C (4–40 °C). NaCl tolerance was assessed using artificial seawater with NaCl concentrations ranging from to 20% in 0.5% increments (0–20%). pH range (pH 5.5 to 9.5, at 0.5 intervals) was tested using specific buffer solutions in marine broth 2216 (MB, Becton Dickinson). Bacterial growth was monitored by measuring OD_600_ values after 1 and 7 days of incubation at 37 °C.

Anaerobic growth conditions (80% N_2_, 10% H_2_, 10% CO_2_) were assessed on MA with or without 1% KNO_3_ supplementation after a 14-day incubation period. Catalase activity was determined by observing gas bubble formation in a 3% H_2_O_2_ solution, while oxidase activity was evaluated using an oxidase reagent kit (bioMérieux SA, Marcy l’Etoile, France) following the manufacturer’s protocol. The bacterial ability to degrade different substrates (alginate, casein, carboxymethylcellulose, DNA, starch, and Tweens) was analyzed according to Qiao et al. [91]. Susceptibility of strain L79^T^ to antibiotics was tested using the disc diffusion method on MA at 37 °C until stable inhibition zones were observed, in accordance with the Clinical and Laboratory Standards Institute (CLSI) guidelines from 2014 [92]. Biomass from strain L79^T^ cultured on MA for 3 days at 37 °C was utilized for API 20E, API 50 CH, and API ZYM assays (bioMérieux). Carbon source oxidation was assessed using BIOLOG GEN III MicroPlates (http://www.biolog.com) accessed on 17 October 2026. All API and BIOLOG experiments were performed concurrently with relevant strains and followed the manufacturer’s instructions.

### 4.5. Functional Validation Experiments

#### 4.5.1. SOX System Validation

For SOX validation experiments, three experimental groups and one control group were established using strains L79^T^, KCTC 72206^T^, and KCTC 72015^T^. The medium contained 3.25 g/L MgSO_4_ as the sulfur source; no additional thiosulfate was added in the control. The medium, devoid of sulfate, consisted of MgCl_2_ (4.83 g/L), CaCl_2_ (1.15 g/L), KCl (0.72 g/L), and NaCl (30 g/L) in artificial seawater. After 5 and 10 days of incubation, 200 μL of the culture solution was transferred to a microporous plate, followed by the addition of 50 μL of ferric chloride and barium chloride separately to observe potential precipitation [93].

#### 4.5.2. ANRA Validation

Assimilatory nitrate reduction experiments involved three experimental groups and one control group with strains L79^T^, KCTC 72206^T^, and KCTC 72015^T^. To minimize interference, the culture medium contained MgCl_2_ (4.83 g/L), MgSO_4_ (3.25 g/L), CaCl_2_ (1.15 g/L), KCl (0.72 g/L), and NaCl (30 g/L) in artificial seawater. Furthermore, 0.5 mM sodium nitrate was supplemented for strain cultivation. Incubation occurred at 25 °C, pH 7.5, and 150 rpm for 48 h in a temperature-controlled shaking incubator. Nitrate was measured using the salicylic acid method at 410 nm; ammonium was measured using the indophenol blue method at 640 nm. Absorbance (OD_600_) readings were taken every 12 h. Nitrate and ammonia levels were determined colorimetrically at the beginning and end of the cultivation period. Quantification of their concentrations in the samples was achieved by measuring absorbance using a spectrophotometer after generating standard curves with nitrate and ammonia standards [94].

#### 4.5.3. Absorption Spectra Assay

For phototrophic experiments, cells were illuminated with cool-white fluorescent lights at an intensity of approximately 40 μmol photons m^−2^ s^−1^ under a 12/12 h light/dark cycle. Strains L79^T^, *P. sediminis* KCTC 72015^T^, and *P. algicola* KCTC 72206^T^ were cultured in the respective liquid medium under dark conditions. After 2 days, a 50 mL cell culture was centrifuged at 900× *g*, and the cells were treated with 3 mL acetone/methanol (7:2) for extraction. Subsequently, the cells were incubated in darkness at room temperature for one hour before measuring the absorption spectra.

### 4.6. Chemotaxonomic Properties

After incubating at 37 °C for 3 days on MA, strain L79^T^ cells were collected by centrifugation and subjected to freeze-drying. The freeze-dried cells were used to extract polar lipids with a specific solvent system (chloroform: methanol: water = 2.5:5:2, *v*/*v*/*v*) following established procedures [95]. The polar lipids were identified through 2D TLC following the protocol outlined by Minnikin et al. [96]. The respiratory quinones of strain L79^T^ were determined using the method detailed by Tindall et al. [97] and analyzed via reverse-phase HPLC as per Hiraishi et al. [98]. Fatty acids from the freeze-dried biomass were extracted, methylated, and analyzed using an Agilent Model 689N gas chromatograph (Agilent Technologies, Santa Clara, CA, USA) and Microbial Identification System (MIDI database: TSBA40) following Cody’s procedure [99].

### 4.7. Transmission Electron Microscopy (TEM) for Observing Structures

To observe the ultrastructure of the photosynthetic membranes in strain L79^T^, transmission electron microscopy (TEM) was employed following established protocols with modifications tailored to bacterial membrane preservation [100]. Cells were cultured under optimal phototrophic conditions (30 °C, light intensity ~40 W/m^2^, aerobic) to maximize membrane development. The cells were harvested during the late exponential phase and initially fixed in 2.5% (*v*/*v*) glutaraldehyde in 0.1 M phosphate buffer (pH 7.2) for 2 h at 4 °C. After washing three times with the same buffer, the cells were post-fixed with 1% (*w*/*v*) osmium tetroxide for 1 h at room temperature. The fixed samples were then dehydrated through a graded ethanol series (50%, 70%, 90%, and 100%) and embedded in Spurr’s resin. Ultrathin sections (70–90 nm) were cut using a Leica Ultracut microtome and collected on copper grids. For enhanced contrast, the sections were stained with 2% uranyl acetate for 10 min followed by Reynolds’ lead citrate for 5 min. The grids were examined using a Hitachi HT-7800 transmission electron microscope (Hitachi High-Tech Corporation, Tokyo, Japan) operating at 100 kV. Images were captured with an AMT XR-16 mid-mount camera (Advanced Microscopy Techniques, Woburn, MA, USA). In parallel, isolated membrane fractions were negatively stained with 2% uranyl acetate on carbon-coated grids to visualize surface details and periodic structures [101]. All TEM procedures were performed in triplicate to ensure reproducibility. However, the resulting images did not have sufficient resolution to clearly resolve the membrane ultrastructure; therefore, these images are not shown in the main text.

### 4.8. Description of Pseudotabrizicola phototrophica sp. nov.

*Pseudotabrizicola phototrophica* [pho.to.tro′phi.ca. Gr. n. *phôs -otos* light; N.L. adj. *trophicus* (from Gr. adj. trophikos) nursing, tending or feeding; N.L. fem. adj. phototrophica feeding on light, phototrophic].

Cells of *Pseudotabrizicola phototrophica* are Gram-stain-negative and short-rod-shaped (0.3–0.5 µm wide and 0.8–1.5 µm long). Colonies appear Faint yellow or opaque-red, circular with entire edges with a diameter of 1.0–1.5 mm. The cell suspension is a light opaque-yellow in color. Cells growth occurs at 4–37 °C (optimum 25–30 °C), with 0.5–4.0% (*w*/*v*) NaCl (optimum 2.5% NaCl) and pH 6.5–9.0 (optimum pH 7.0–7.5). Phototrophic growth occurs under aerobic, heterotrophic conditions, and photosynthetic pigments are produced. It is positive for oxidase activity, peroxidase activity and nitrate reduction and is unable to hydrolyze agar, sodium alginate, CM-cellulose, starch, Tween20, Tween40, Tween60, Tween80, DNA, and casein. The dominant fatty acids (>10%) are Summed Feature 8 (C_18:1_ω7c/C_18:1_ω6c) and iso-C_18:0_. The main respiratory quinone is Q-10. The polar lipids consisted of phosphatidylethanolamine (PE), phosphatidylglycerol (PG), phosphati-dylcholine (PC) and three unidentified lipids (L_1_, L_2_, L_3_).

The type strain, L79^T^ (=KCTC 8007^T^ = MCCC 1H01415^T^ = SDUM073006^T^), was isolated from intertidal sediments of Xiaoshi Island, Yellow Sea of Weihai, People’s Republic of China (122°1′ E, 37°31′ N). The DNA G + C content of type strain was 62.3 mol%. The Genebank accession number for the 16S rRNA gene sequence of strain L79^T^ was PP544386 and the draft genome has been deposited in GenBank under the accession number JAZHDA010000000.

## Figures and Tables

**Table 1:** Distribution of key metabolic genes among *Pseudotabrizicola* species. Strains: 1. L79^T^ (data from this study); 2. *P. algicola* KCTC 72206^T^; 3. *P. sediminis* KCTC 72015^T^. +, present; −, absence. Only three strains with complete genome data were included in this comparative analysis.

Gene/Pathway	Function	1	2	3
**Photosynthesis**				
*pufL*	Reaction center L subunit	+	+	+
*pufM*	Reaction center M subunit	+	+	+
*pufH*	Reaction center H subunit	+	+	+
*bch* cluster	Bacteriochlorophyll biosynthesis	+	+	+
**PHB metabolism**				
*phbB*	Acetoacetyl-CoA reductase	+	+	+
*phaC*	PHB synthase subunit C	+	+	+
*phaE*	PHB synthase subunit E	+	+	+
*phaZ*	PHB depolymerase	+	+	+
*phaR*	PHB synthesis repressor	+	+	+
**Thiosulfate oxidation**				
*sox* cluster (soxABCDXYZ)	SOX system	+	−	+
**Sulfate assimilation**				
*cys* genes	Assimilatory sulfate reduction	+	−	−
**Nitrate assimilation**				
*nas*	Assimilatory nitrate reductase	+	+	+
*nir*	Assimilatory nitrite reductase	+	+	+

**Table 2:** Secondary metabolite biosynthetic gene clusters predicted in the genome of strain L79^T^ by antiSMASH.

Cluster	Type	Most Similar Known Cluster	Similarity Score(%)	Compound (s)
1	Terpene	Carotenoid	82	Carotenoids
2	NRPS	Pyreudione	74	Pyreudione A/B/C/D/E
3	PKS	Unknown	60	6-methylsalicyclic acid
4	Terpene-precursor	Unknown	49	Sodorifen
5	Butyrolactone	(2R,3S,4S)-5-fluoro-2,3,4-trihydroxypentanoic acid	46	Putative butyrolactone-type signaling molecule
6	Ectoine	Ectoine	43	Partial ectoine-related gene cluster (ectoine utilization potential)
7	RiPP-like	Unknown	28	Putative bufferin-like metallophore

**Table 3:** Comparison of the carotenoid biosynthetic gene cluster in strain L79^T^ with known carotenoid clusters from related bacteria. Note: The genes listed in this table are specifically involved in carotenoid biosynthesis and are distinct from the chlorophyll biosynthesis and LHC genes, which are not included here. +, present; −, absence.

Gene	Function	L79^T^	*P. algicola* KCTC 72206^T^	*P. sediminis* KCTC 72015^T^
*crtE*	Geranylgeranyl pyrophosphate synthase	+	+	+
*crtB*	Phytoene synthase	+	+	+
*crtI*	Phytoene desaturase	+	+	+
*crtC*	Acyclic carotenoid 1,2-hydratase	+	+	+
*crtD*	1-hydroxycarotenoid 3,4-desaturase	+	+	+
*crtF*	Demethylspheroidene O-methyltransferase	+	+	+
*crtY*	Lycopene cyclase	−	−	−
*crtZ*	β-carotene hydroxylase	−	−	−
*crtW*	Carotenoid ketolase	−	−	−

**Table 4:** SOX pathway validation experiment results. − indicates no obvious precipitation; + indicates precipitation; ++ indicates much precipitation.

Day0/ Day5/ Day10	Control			FeCl_3_			BaCl_2_		
Control	−/−/−	−/−/−	−/−/−	−/−/−	−/−/−	−/−/−	−/−/−	−/−/−	−/−/−
	−/−/−	−/−/−	−/−/−	−/−/−	−/−/−	−/−/−	−/−/−	−/−/−	−/−/−
	−/−/−	−/−/−	−/−/−	−/−/−	−/−/−	−/−/−	−/−/−	−/−/−	−/−/−
L79^T^	−/−/−	−/−/−	−/−/−	−/−/−	−/−/−	−/−/−	−/++/+	−/++/+	−/++/+
	−/−/−	−/−/−	−/−/−	−/−/−	−/−/−	−/−/−	−/++/+	−/++/+	−/++/+
	−/−/−	−/−/−	−/−/−	−/−/−	−/−/−	−/−/−	−/++/+	−/++/+	−/++/+

## Data Availability

The datasets presented in this study can be found in online repositories. The Genebank accession number for the 16S rRNA gene sequence of strain L79^T^ was PP544386 (https://www.ncbi.nlm.nih.gov/nuccore/PP544386.1/) accessed on 17 October 2026 and the draft genome has been deposited in GenBank under the accession number JAZHDA010000000 (https://www.ncbi.nlm.nih.gov/nuccore/JAZHDA000000000.1/) accessed on 17 October 2026.

## Supplementary Materials

The following supporting information can be downloaded at: https://www.mdpi.com/article/10.3390/md24100364/s1, Figure S1: Maximum likelihood tree showing phylogenetic relationships based on the 16S rRNA gene sequences of L79^T^ with closely related species of the genus *Pseudotabrizicola*. *Roseobacter litoralis* ATCC 49566^T^ was used as the outgroup. Bootstrap values above 70% (1000 replicates) are shown at branch nodes. Bar, 0.05 substitutions per nucleotide position; Figure S2: Phylogenomics tree showing phylogenetic relationships based on the protein sequences of strain L79^T^ and closely related members of the family *Rhodobacteraceae*. Bootstrap values are based on 1000 replicates; only bootstraps exceeding 70% are shown. Bar, genetic distance of 0.05. *Roseobacter litoralis* ATCC 49566^T^ was used as an outgroup. GenBank assembly accession numbers are provided in parentheses; Table S1: Differential characteristics between strain L79^T^ and the reference *Pseudotabrizicola* strains. Strains: 1. L79^T^ (data from this study); 2. *P. algicola* KCTC 72206^T^; 3. *P. sediminis* KCTC 72015^T^. Data for *P. algicola* KCTC 72206^T^ and *P. sediminis* KCTC 72015^T^ are from Park et al. (2020) and Liu et al. (2019), respectively [27,29]. All other data are from this study; Table S2: The physiological and biochemical features of strain L79^T^ compared with those of phylogenetically related members of the genus *Pseudotabrizicola*. Strains: 1. L79^T^ (data from this study); 2. *P. algicola* KCTC 72206^T^; 3. *P. sediminis* KCTC 72015^T^. +, positive; −, negative; w, weak. All data were from this study unless indicated otherwise; Table S3: Genomic characteristics of all strains and ANI/DDH values between strains of genus *Pseudotabrizicola* and strain L79^T^. Strains: 1, L79^T^; 2, *P. algicola* KCTC 72206^T^; 3, *P. alkalilacus* KCTC 62173^T^; 4, *P. sediminis* KCTC 72015^T^; 5, *P. formosa* KCTC 52636^T^; Table S4: The key genes/protein predicted to be involved in PHB catabolism in strain L79^T^.
