# Supplementary material for: Metabolic Versatility and Bioactive Metabolite Potential of Pseudotabrizicola phototrophica sp. nov., an Aerobic Anoxygenic Phototroph from Intertidal Sediments
Source: Mar Drugs. 2026 Oct 19;24(10):364. doi: 10.3390/md24100364 (PMC13706104; doi:10.3390/md24100364)
Supplement: Supplementary file 1 [file marinedrugs-24-00364-s001.zip › marinedrugs-4542195-supplementary.pdf]

Metabolic versatility and bioactive metabolite potential of *Pseudotabrizicola phototrophica* sp. nov., an aerobic anoxygenic phototroph from intertidal sediments

Li Hu[§a], Meng-Qi Ye[§* a, b, c], Jin-Hao Xu [a], Yu-Qi Ye [a], Xin-Yun Tan [a] , Zong-Jun Du[*a,b,c]

[a] Marine College, Shandong University, Weihai, Shandong, 264209, PR China.

[b] State Key Laboratory of Microbial Technology, Qingdao, 266237, PR China

[c] Weihai Research Institute of Industrial Technology of Shandong University, Weihai, 264209, PR China

[§] These two authors contributed equally to this work.

*** Correspondence author：**

yemengqi@sdu.edu.cn（M.-Q.-Y）

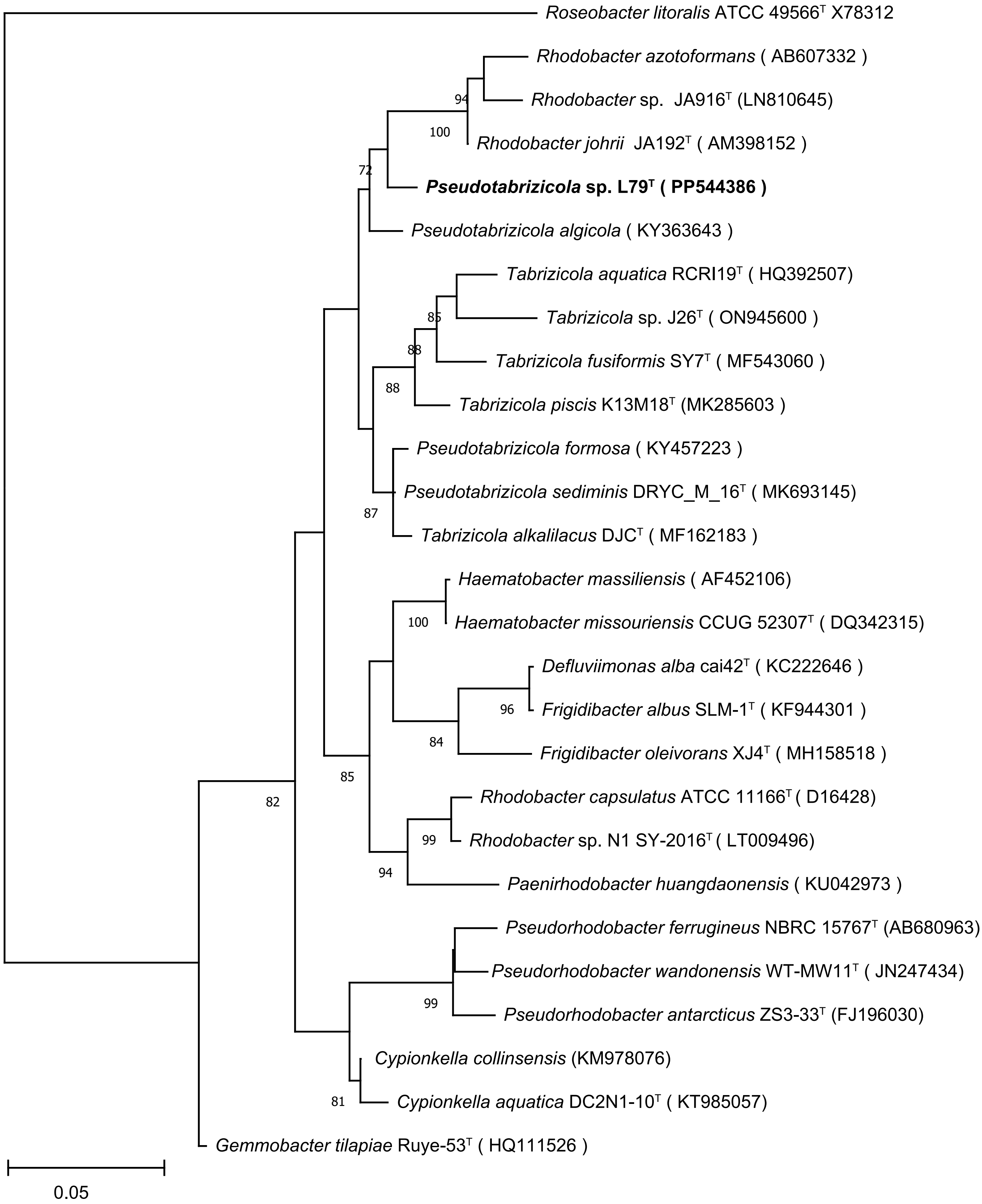

Figure S1. Maximum likelihood tree showing phylogenetic relationships based on the 16S rRNA gene sequences of L79^{T} with closely related species of the *genus Pseudotabrizicola*. *Roseobacter litoralis* ATCC 49566^{T} was used as the outgroup. Bootstrap values above 70% (1,000 replicates) are shown at branch nodes. Bar, 0.05 substitutions per nucleotide position.

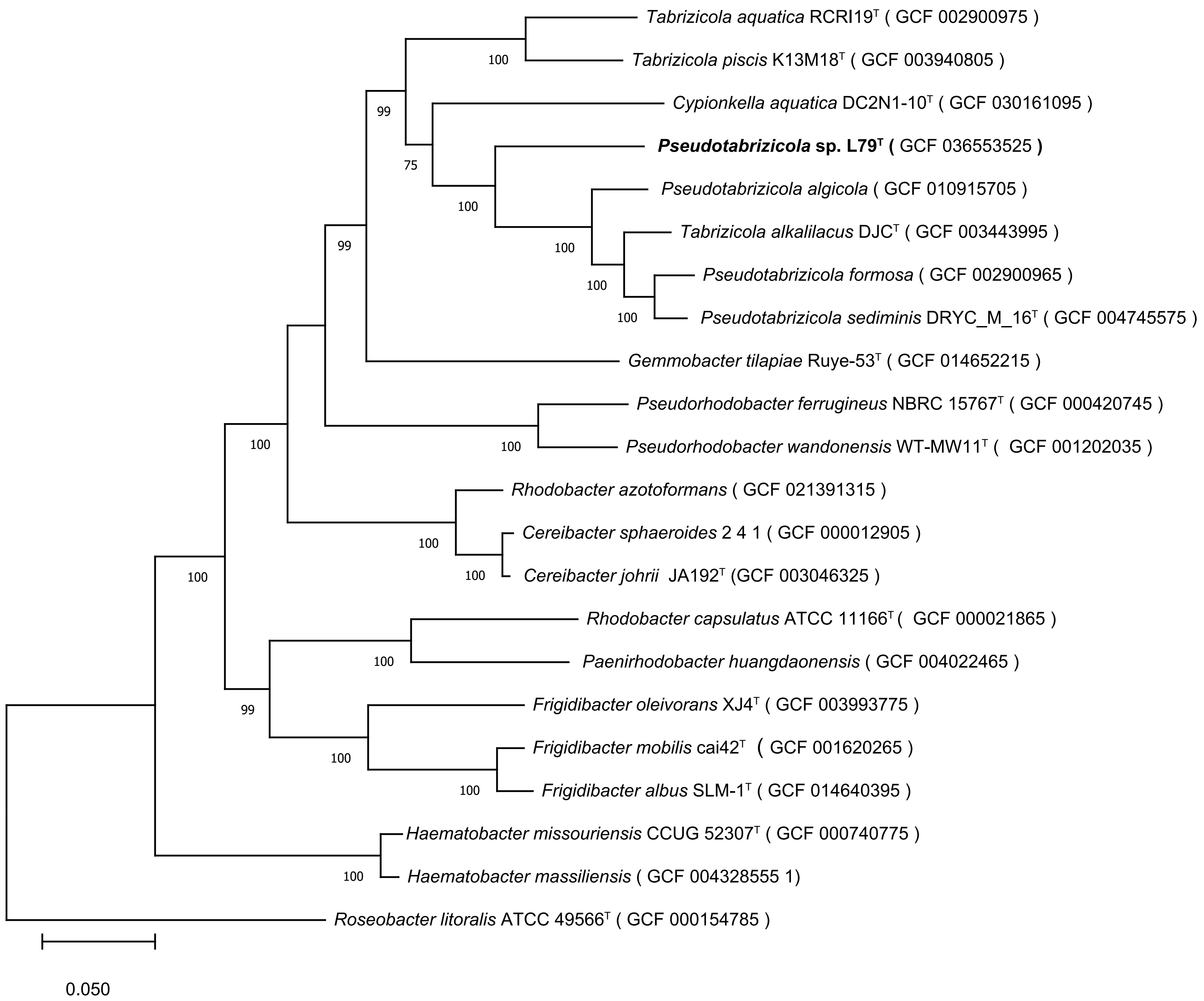

Figure S2. Phylogenomics tree showing phylogenetic relationships based on the protein sequences of strain L79^T and closely related members of the family *Rhodobacteraceae*. Bootstrap values are based on 1000 replicates; only bootstraps exceeding 70% are shown. Bar, genetic distance of 0.05. *Roseobacter litoralis* ATCC 49566^T was used as an outgroup. GenBank assembly accession numbers are provided in parentheses.

Table S1. Differential characteristics between strain L79^T and the reference *Pseudotabrizicola* strains. Strains: 1. L79^T (data from this study); 2. *P.algicola* KCTC 72206^T ; 3. *P.sediminis* KCTC 72015^T. Data for *P. algicola* KCTC 72206^T and *P. sediminis* KCTC 72015^T are from Park et al. (2020) and Liu et al. (2019), respectively. All other data are from this study.

Characteristic	1	2	3
Colour	Faint yellow; opaque-red (Light culture for two weeks)	Cream	Opaque-pink
Isolation	Intertidal sediments	Microalga culture	Saline lake Sediment
Temperature (optimum, °C)	25-30	25–37	20-25
NaCl (optimum, %, w/v)	2.5	2.0	1.5
pH (optimum)	7.0–7.5	7.0–8.5	7.0–7.5
**Enzyme activity:**			
Nitrate reduction	+	+	-
Oxidase	+	+	+
Catalase	+	+	+
Urease	-	-	+
**Hydrolysis of:**			
Starch	-	+	-
Gelatin	-	+	-
phycocolloid	-	-	-
casein	-	-	-
Tween 20	-	-	-
Tween 40	-	-	-
Tween 60	-	-	-
Tween 80	-	-	-
**Major fatty acids (≥10%)**	Summed Feature 8 ($C_{18:1}$-*ω7c*/$C_{18:1}$-*ω6c*) and iso-$C_{18:0}$	$C_{18:1}$-*ω6c* and iso-$C_{18:0}$	Summed Feature 8 ($C_{18:1}$-*ω7c*/$C_{18:1}$-*ω6c*) and iso-$C_{18:0}$
**Polar lipids**	PG,PE,PC,Ls	PG,PE,PC,i-PE,Als ,Ls	PC, PG, DPG, PE, APL, AL, PL, L
**Major quinone**	Q-10	Q-10	Q-10

Table S2. The physiological and biochemical features of strain L79^T compared with those of phylogenetically related members of the genus *Pseudotabrizicola.*

Strains: 1. L79^T (data from this study); 2. *P.algicola* KCTC 72206^T ; 3. *P.sediminis* KCTC 72015^T.

+, positive; −, negative; w, weak. All data were from this study unless indicated otherwise.

	1	2	3
Stachyose	-	+	+
β-Methyl-D-Glucose	+	+	-
N-acetyl-D-Glucosamine	+	+	-
D-Fucose	+	+	-
D-Mannitol	+	+	-
D-Sorbitol	+	-	-
D-Fructose-6-PO_4	+	+	-
D-Galacturonic acid	+	+	+
D-Glucuronic acid	+	+	+
Glucuronamide	-	+	+
Raffinose	-	+	+
α-D-Glucose	+	-	+
Quinic acid	-	-	-
Methyl pyruvate	+	+	+
Citric acid	+	+	+
α – Hydroxy-butyric acid	+	+	+
Alkaline phosphatase	+	-	+
Valine arylamidase	-	+	+
α-galactosidase	-	-	+
β-galactosidase	-	-	+
N-acetyl-β- glucosaminidase	+	+	-
2-Nitrophenyl β-D-galactopyranoside	+	+	+
phenylalanine deaminase	w	w	w
raffinose	+	+	+
L-arabinose	-	+	-
Glycol	-	-	-
Sorbitol	-	-	-
Stachyose	-	+	+

Table S3. Genomic characteristics of all strains and ANI/DDH values between strains of genus *Pseudotabrizicola* and strain L79^T. Strains: 1, L79^T; 2, *P. algicola* KCTC 72206^T; 3, *P. alkalilacus* KCTC 62173^T; 4, *P. sediminis* KCTC 72015^T; 5, *P. formosa* KCTC 52636^T.

	1	2	3	4	5
Size(bp)	3,950,661	4,491,281	4,610,061	4,040,697	3,702,758
Contigs	58	29	110	86	14
N50 length (bp)	363,760	694,770	284,500	292,200	812,613
GC% content (mol %)	62.3	64.4	62.9	63.0	63.5
Genes	3880	4373	4470	3902	3552
Protein-coding genes	3804	4262	4413	3714	3470
rRNA genes	3	3	6	3	3
tRNA genes	48	46	48	42	46
ncRNA genes	3	3	3	3	3
ANI/DDH(%)	-	77.1/23.1	76.7/22.6	76.5/23.1	76.8/22.5

Table S4. The key genes/protein predicted to be involved in PHB catabolism in strain L79^T.

Gene/Protein ID	Gene/Protein name	Annotation
K03821	*pha*C/*phb*C	Poly[(R)-3-hydroxyalkanoate] polymerase subunit
K22881	*pha*E	Poly[(R)-3-hydroxyalkanoate] polymerase subunit
K05973	*pha*Z	Poly(3-hydroxybutyrate) depolymerase
K00023	*phb*B	Acetoacetyl-CoA reductase
ABA79555	PhaR	Polyhydroxyalkanoate synthesis repressor
